# Preparation of a Mesoporous Biosensor for Human Lactate Dehydrogenase for Potential Anticancer Inhibitor Screening

**DOI:** 10.1021/acsbiomaterials.3c00582

**Published:** 2023-10-19

**Authors:** Clarissa Cocuzza, Elena Antoniono, Carminna Ottone, Valentina Cauda, Debora Fino, Marco Piumetti

**Affiliations:** † Department of Applied Science and Technology, Politecnico di Torino, Corso Duca degli Abruzzi, 24, 10129 Turin, Italy; ‡ Escuela de Ingeniería Bioquímica, Pontificia Universidad Católica de Valparaíso, Av. Brasil 2085, Valparaíso 2340000, Chile

**Keywords:** human lactate dehydrogenase, *h*LDH-A, covalent attachment, enzyme immobilization, MCM-41, mesoporous silica, NHI-2

## Abstract

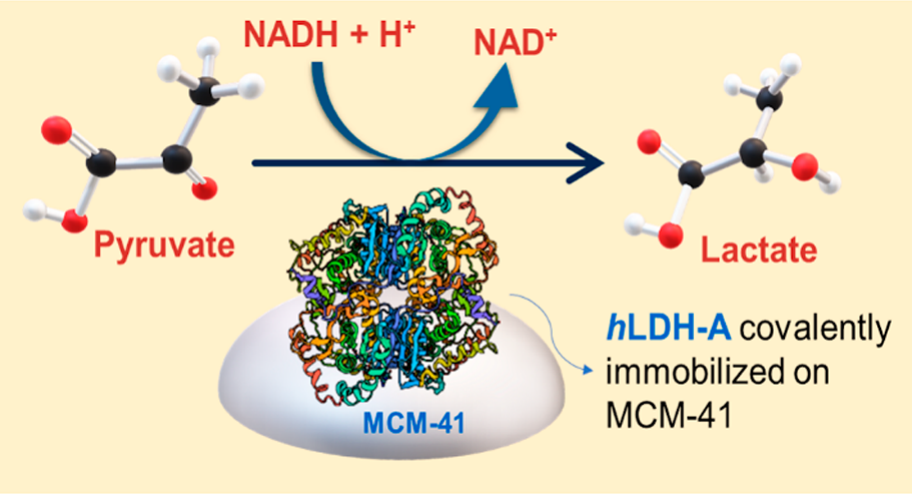

Cancer is the second leading cause of death worldwide, with a dramatic impact due to the acquired resistance of cancers to used chemotherapeutic drugs and treatments. The enzyme lactate dehydrogenase (LDH-A) is responsible for cancer cell proliferation. Recently the development of selective LDH-A inhibitors as drugs for cancer treatment has been reported to be an efficient strategy aiming to decrease cancer cell proliferation and increase the sensitivity to traditional chemotherapeutics. This study aims to obtain a stable and active biocatalyst that can be utilized for such drug screening purposes. It is conceived by adopting human LDH-A enzyme (*h*LDH-A) and investigating different immobilization techniques on porous supports to achieve a stable and reproducible biosensor for anticancer drugs. The *h*LDH-A enzyme is covalently immobilized on mesoporous silica (MCM-41) functionalized with amino and aldehyde groups following two different methods. The mesoporous support is characterized by complementary techniques to evaluate the surface chemistry and the porous structure. Fluorescence microscopy analysis confirms the presence of the enzyme on the support surface. The tested immobilizations achieve yields of ≥80%, and the best retained activity of the enzyme is as high as 24.2%. The optimal pH and temperature of the best immobilized *h*LDH-A are pH 5 and 45 °C for the reduction of pyruvate into lactate, while those for the free enzyme are pH 8 and 45 °C. The stability test carried out at 45 °C on the immobilized enzyme shows a residual activity close to 40% for an extended time. The inhibition caused by NHI-2 is similar for free and immobilized *h*LDH-A, 48% and 47%, respectively. These findings are significant for those interested in immobilizing enzymes through covalent attachment on inorganic porous supports and pave the way to develop stable and active biocatalyst-based sensors for drug screenings that are useful to propose drug-based cancer treatments.

## 1 Introduction

**Figure 1:**
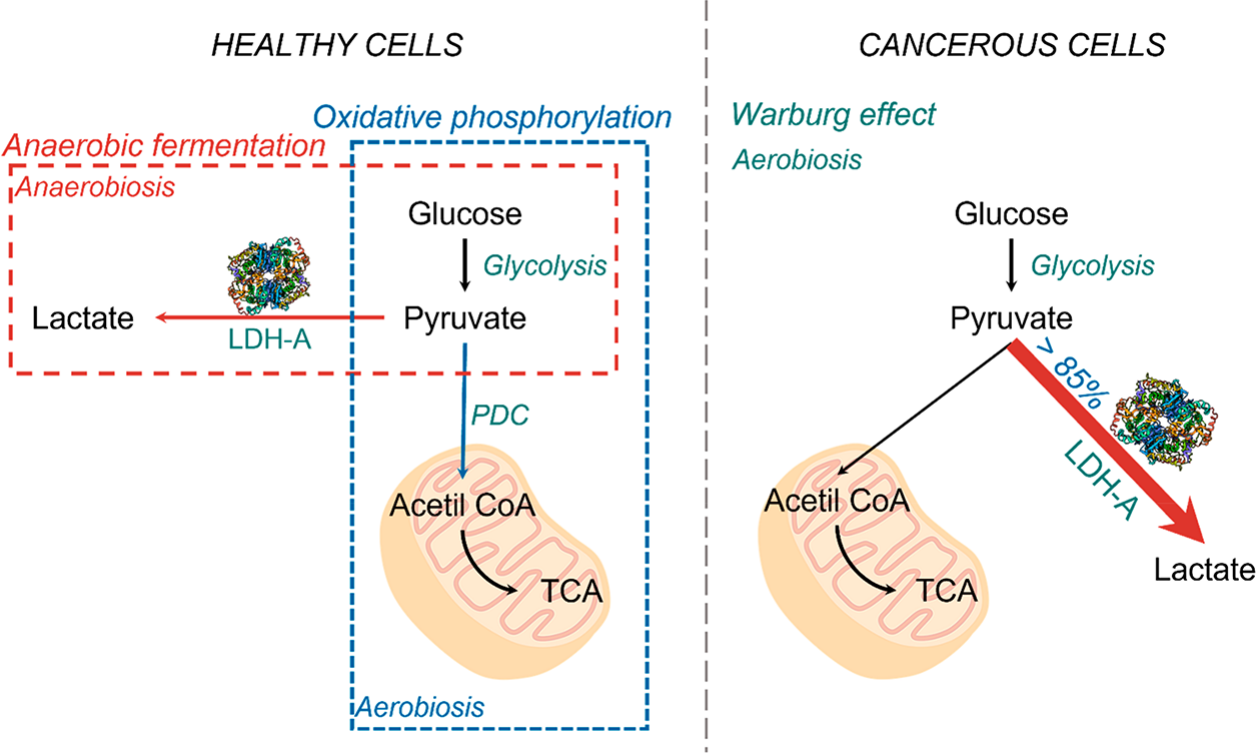
Scheme of the Warburg effect in cancer cells in comparison with glycolysis in healthy cells adapted from ref (9) (the enzyme structure was created with BioRender.com).

According to the *International Agency for Research on Cancer* (IARC) survey, in 2040 new cancer cases are expected to increase to 30.2 million and the number of deaths is assumed to become 16.3 million.^1,2^ The most common are lung, bronchus, colorectal, breast, prostate, and finally tracheal cancers. Moreover, pancreatic cancer is one of the most lethal.^3^ The cells of all these cancer typologies are subjected to aerobic glycolysis or Warburg effect.^3−9^ As a whole, cancer cells metabolize glucose into pyruvic acid and then convert it to lactate, even in the presence of oxygen. As schematized in Figure 1, healthy cells transform pyruvate into lactate only under hypoxic conditions. By contrast, cancerous cells metabolize glucose to pyruvic acid, which is then converted to lactate even in aerobic conditions.^9^ Cancer cells take advantage of this accelerated fermentation process^10^ to obtain the large amount of necessary energy (ATP molecules) and the required precursors (such as nucleic acid, proteins, and lipids) for cell proliferation.^11^

**Figure 2:**
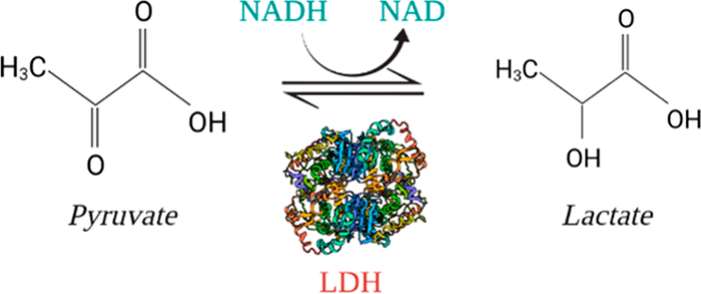
Scheme of the reaction catalyzed by *h*LDH-A (the enzyme structure was created with BioRender.com).

Lactate dehydrogenase (LDH) is the enzyme that catalyzes the conversion of pyruvate into lactate. LDH-A is the isoform of lactate dehydrogenase which is overexpressed in cancer cells.^4^ It is fundamental for cancer cell proliferation because it preferentially converts pyruvate into lactate.^8,12^ This reaction regenerates the NAD^+^ necessary for glycolysis and produces lactate that behaves as a signaling molecule, allowing aerobic glycolysis to occur.^11,13^ Due to the role of lactate dehydrogenase in cancer cell proliferation, in recent years there has been an increasing interest in the inhibition of LDH-A. The inhibition of this enzyme seems to be a promising strategy for chemotherapy.^9^ In fact, the development of selective LDH-A inhibitors as drugs for cancer treatment aims to obtain chemotherapeutic agents, which are able to decrease cancer cell proliferation ability and have less serious side effects than traditional chemotherapies. In addition, LDH-A inhibition increases the sensitivity of cancer to traditional chemotherapeutic agents.^14,15^Figure 2 provides a scheme of the reaction catalyzed by *h*LDH-A, in the presence of inhibitors, the production of lactate is hampered.

Currently, the only procedure to test the efficiency of chemical compounds in inhibiting enzymes is to perform an enzymatic assay for each individual molecule.^16^ During the screening phase, several thousand compounds are typically tested. For example, a study carried out in 2019, by Zhou et al.,^17^ tested about 5000 molecules to develop new LDH-A inhibitors. These facts imply the necessity of high amounts of enzyme and therefore high costs. In order to reduce the cost of this screening phase, the enzyme should be recovered after each test. The immobilization of enzymes on solid supports is a possible strategy to simplify continuous operations because it allows recovering the enzyme from the reaction solution avoiding its disposal and reducing the overall cost.^18,19^

As a whole, different strategies for immobilization have been proposed over the years. These can be divided into reversible (adsorption and formation of disulfide bonds) and irreversible (covalent coupling, entrapment, and cross-linking) methods.^20^ To obtain a stable and durable biocatalyst, irreversible methods are more appropriate, because the strength of the formed bonds provides higher stability than the reversible techniques. Among the possible irreversible immobilization techniques, covalent binding provides the least enzyme leakage and potentially the greatest stabilization.^20^ To perform a covalent coupling, the used support must present proper functional groups (such as amino and aldehyde groups) that react with the amino acid residues of the enzyme to form covalent bonds.

Mesoporous silicas have shown great potential as ideal materials for the covalent immobilization of enzymes.^21^ In fact, mesoporous silica such as MCM-41, MCF, and SBA-15 possess high specific surface areas^22^ and pore volumes, as well as large pores, good mechanical and chemical stability in aqueous media, and tunable morphology, along with the possibility of being activated postsynthesis with different functional groups (such as amino, aldehyde, or epoxy groups) to form the support–enzyme covalent bond.^21,23^

In this work, the human lactate dehydrogenase enzyme (*h*LDH-A) was immobilized through covalent coupling methods on functionalized mesoporous silica to obtain *h*LDH-A immobilized on MCM-41 (imm-*h*LDH-A) to evaluate if it could be used as a biosensor for the screening of *h*LDH-A inhibitors. This device could accelerate the test of drugs’ efficiency and would allow the recovery of the enzyme, with a consequent reduction in the cost of the screening phase. To the best of the authors’ knowledge, this is the first time that human lactate dehydrogenase is immobilized on mesoporous silica to realize a biosensor for the test of *h*LDH-A inhibitors. Amino-aldehyde and amino-MCM-41 are used to immobilize *h*LDH-A using two different techniques that form covalent bonds between the enzyme and the support. The resulting imm-*h*LDH-A samples were characterized by retained activity (*R*_act_) and the immobilization yield (IY). The activity of the best performing imm-*h*LDH-A was studied by varying the values of pH and temperature, along with its thermal stability. The results obtained were compared to those obtained for free *h*LDH-A. Finally, some preliminary tests were carried out to evaluate the feasibility of using imm-*h*LDH-A as a catalyst in a LDH-based biosensor. The reusability of the imm-*h*LDH-A was investigated through a 4-cycle batch reaction. Then, the reliability of the inhibition tests performed on imm-*h*LDH-A was studied by comparing it with the inhibition efficiency achieved on the free *h*LDH-A.

## 2 Materials and Methods

### 2.1 Materials

MCM-41, (3-glycidyloxypropyl)trimethoxysilane (GPTMS), (3-aminopropyl)triethoxysilane (APTES), sulfuric acid, hydrochloric acid (37% wt.), poly(ethylene glycol), toluene, acetone, CuSO_4_, KH_2_PO_4_, K_2_HPO_4_, NaHCO_3_, Na_2_CO_3_, H_2_NaO_4_P, Na_2_HPO_4_, 1-hydroxy-6-phenyl-4-(trifluoromethyl)-1*H*-indole-2-carboxylic acid methyl ester (NHI-2), dimethyl sulfoxide (DMSO), and l-lactate dehydrogenase from human expressed in *E. coli* (*h*LDH-A, EC 1.1.1.27) were supplied from Sigma-Aldrich (Merck). Potassium iodate, glutaraldehyde (50% in water solution), sodium meta periodate, lactic acid, sodium pyruvate, sodium borohydride, NAD, and NADH were purchased from VWR avantor.

### 2.2 Methods

#### 2.2.1 Activity Assay

The activity tests were performed using a UV–vis spectrophotometer (Jasco V-730) by monitoring for 60 s the decrease in absorbance at 340 nm, which corresponds to the peak of NADH. The absorbance variation in time (Δ*A*) can be correlated with the specific activity, of the free (*A*_FE_, U mg_prot_^–1^) or immobilized enzyme (*A*_IE_, U g_supp_^–1^), using eq 1 and eq 2, respectively.
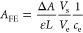
1
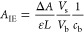
2where Δ*A* is the absorbance slope, ε is the NADH molar extinction coefficient (6.22 mM^–1^ cm^–1^), *L* is the optical path (cm), *V*_s_ is the volume of the solution in the cuvette (mL), *V*_e_ and *V*_b_ are the volume of enzymatic solution and suspension of the imm-*h*LDH-A, respectively (mL), and *c*_e_ and *c*_b_ are the concentration of the enzymatic solution (mg mL^–1^) and suspension of the imm-*h*LDH-A (g mL^–1^), respectively. In order to measure the enzyme’s activity, 100 μL of *h*LDH-A solution (0.01 mg mL^–1^, according to Bradford assay^24^) or 100 μL of imm-*h*LDH-A suspension (20 mg mL^–1^) was added to the solution present in the cuvette for analysis containing 100 μL of 7 mM cofactor (NADH) solution (in 0.1 M phosphate buffer, pH 7.5) and 100 μL of 49 mM pyruvate solution (in 0.1 M phosphate buffer, pH 7.5) dispersed in 2.7 mL of 0.1 M phosphate buffer, pH 7.5, kept at 35 °C.

#### 2.2.2 Kinetic Studies of Lactate Dehydrogenase

*h*LDH is a tetrameric enzyme that presents five main isoforms composed of four subunits of type A or B.^9^ The two principal isoforms are homotetramers: *h*LDH-A and *h*LDH-B.^9^ The first preferentially converts the pyruvate into lactate, while LDH-B mediates the reverse reaction.^4,8^ To examine the different affinity of free *h*LDH-A toward pyruvate and lactate, the kinetic parameters of these two reactions were studied. For the pyruvate reduction, the analysis was performed following the same procedure used for the activity assay, varying the concentration of the substrate solution from 0.1 to 30 mM (the pyruvate concentration in the cuvette of analysis ranges from 0.003 to 1 mM). The kinetic parameters were obtained through the Hanes–Woolf linearization for the pyruvate reduction.^25^ To study the kinetic parameters of the lactate oxidation, the enzymatic assays were performed at 45 °C adding 100 μL of *h*LDH-A (0.01 mg mL^–1^) to a solution formed by 2.7 mL of 0.1 M phosphate buffer, pH 8, 100 μL of 20 mM cofactor (NAD) solution (in 0.1 M phosphate buffer, pH 8), and 100 μL of the substrate (lactate) solution (in 0.1 M phosphate buffer pH 8) with concentrations from 0.5 to 100 mM (the lactate concentration in the cuvette for analysis ranges from 0.02 to 2.67 mM).

#### 2.2.3 Support Functionalization

##### 2.2.3.1 MCM-41 Activated with Amino Functional Groups (MCM-41_A_)

To functionalize the mesoporous silica with amino groups, 1 g of MCM-41 was put in contact with 30 mL of APTES solution (APTES 5% v/v in toluene) and then vigorously stirred at 105 °C for 5 h.^26^ The support was filtered and washed with acetone (30 mL) and water at the end of the reaction, and finally it was dried at room temperature. A scheme of the functionalization process is displayed in Figure S1. The moles of amino groups grafted on the silica surface were estimated through the adsorption of CuSO_4_ on -NH_2_.^27,28^ The amount of CuSO_4_ in the solution was analyzed through UV–vis spectroscopy and the moles of amino groups were calculated by eq 3:
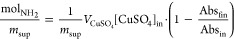
3where mol_NH_2__ are the moles of amino groups (mmol), *m*_sup_ is the amount of characterized MCM-41_A_ (g), *V*_CuSO_4__ is the volume of copper sulfate solution (mL), [CuSO_4_]_in_ is the concentration of CuSO_4_ in the solution (mmol mL^–1^), and Abs_in_ and Abs_fin_ are the values of absorbance measured at 750 nm for the supernatant at the beginning and the end of the reaction.

##### 2.2.3.2 MCM-41 Activated with Amino and Glyoxyl Functional Groups (MCM-41_AG_)

To have both amino and glyoxyl groups, the MCM-41 was functionalized with a three-step procedure, following the method reported by Cocuzza et al., and the process is schematized in Figure S1.^29^ The first phase is the same used for the activation with amino groups, with the addition of GPTMS to the solution: 1 g of MCM-41 was put in contact with 30 mL of APTES and GPTMS solution (APTES 5% v/v and GPTMS 5% v/v in toluene) and strongly stirred at 105 °C for 5 h. The support was filtered and washed with acetone in the same amount of toluene and abundant distilled water. At the end of the first step, MCM-41 was functionalized with amino and epoxy groups. The epoxy groups were hydrolyzed during the second phase: the material obtained (1 g) was put in contact with 0.1 M H_2_SO_4_ (30 mL) and vigorously stirred for 2 h at 85 °C. The material obtained was filtered and washed with abundant distilled water. At the end of this phase, the epoxy group was hydrolyzed to two diols. During the last step, the modified MCM-41 (1 g) was put in contact with 0.1 M NaIO_4_ (30 mL) and vigorously stirred for 2 h at room temperature. In this phase, the diols are oxidized to obtain glyoxyl groups. At the end of the reaction, MCM-41_AG_ was filtered, washed with abundant distilled water, and dried at room temperature. The number of glyoxyl groups on the silica surface was quantified through the back-titration method with NaHCO_3_/KI.^26^ The moles of glyoxyl groups were determined by eq 4:
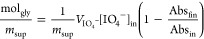
4where mol_gly_ are the moles of glyoxyl groups (mmol), *m*_sup_ is the amount of functionalized MCM-41 (g), *V*_IO_4_^–^_ is the volume of metaperiodate solution (mL), [IO_4_^–^] is the concentration of IO_4_^–^ ions in the NaIO_4_ solution (mmol mL^–1^), and Abs_in_ and Abs_fin_ are the values of absorbance measured at 420 nm for the supernatant at the beginning and the end of the reaction, respectively.

#### 2.2.4 Support Characterization

The specific surface area (*S*_BET_) and the micropore volume (*V*_p_) of the unmodified MCM-41, MCM-41_A_, and MCM-41_AG_ were estimated by N_2_ physisorption at −196 °C, with Micromeritics ASAP TriStar II 3020 instrument, after pretreatment at 200 °C for 2 h. The specific surface areas were calculated by applying the Brunauer–Emmet–Teller method, and the micropore volumes were evaluated by applying the Barrett–Joyner–Halenda approach to the desorption phase.

The morphology of the samples was investigated through high-resolution transmission electron microscopy (TEM, Jeol JEM 3010 UHR, LaB_6_ gun, operating at 200 kV). The microscopies obtained were analyzed with Gatan software.

Powder X-ray diffractograms of MCM-41, MCM-41_A_, and MCM-41_AG_ were acquired with an EMPYREAN diffractometer, Cu Kα radiation, 2θ range of 1°–5°, angle step size 0.013, and 120 s time per step. The diffractograms were examined using the Powder Data File Databases (PDF-2 Release 2004, COD_Mar10).

To verify that MCM-41 was properly functionalized, Fourier transform IR (FT-IR) spectra were collected on the unmodified support, MCM-41_A_, and MCM-41_AG_. The IR spectra were acquired with a Bruker INVENIO instrument equipped with liquid nitrogen cooled MCT detector. The samples were pretreated at 300 °C in standard vacuum conditions for 1 h, and the analyses were performed at room temperature (range 4000–400 cm^–1^, 64 scans, resolution 4 cm^–1^).

#### 2.2.5 Immobilization of *h*LDH-A on Support

##### 2.2.5.1 Immobilization on MCM-41_A_ Preactivated with Glutaraldehyde (MCM-41_A_-GA)

To immobilize *h*LDH-A on MCM-41_A_, the support was preactivated with glutaraldehyde, adapting the procedure reported by Alagöz et al. (a detailed scheme of the immobilization is reported in Figure S2a).^30^ The support (1 g) was incubated in 25 mL of glutaraldehyde solution (1 or 2.5% v/v in 50 mM sodium phosphate, pH 7) and kept under vigorous stirring at room temperature for 2 h. At the end of the preactivation step, the support was filtered, washed with distilled water, and immediately used to immobilize *h*LDH-A. Two milligrams of *h*LDH-A (according to Bradford assay) was put in contact with 1 g of MCM-41_A_-GA in 40 mL of 25 mM sodium phosphate, pH 7,^31^ keeping the mixture at 10 °C under gentle stirring. Regularly samples were collected to estimate the immobilization time by monitoring the activity of the supernatant. To check whether the enzyme was inactivated by the immobilization conditions, the activity of the suspension and the activity of an enzymatic solution, which was kept under the same conditions as the immobilizing mixture but not put in contact with the support (used as a blank), were monitored. The immobilization ended when the supernatant activity dropped to zero or remained constant for two consecutive measurements. The solution was filtered under vacuum and washed with 25 mM phosphate buffer, pH 7.5, and a sample of the filtered solution was collected to monitor if the phosphate buffer caused the enzyme leakage. Finally, the imm-*h*LDH-A was washed with distilled water and dried at 4 °C.

##### 2.2.5.2 Immobilization on MCM-41_AG_

The immobilization of *h*LDH-A, schematized in Figure S2b, was performed by adapting the procedures described by Jackson et al.^32^ and Cocuzza et al.^29^ The immobilization was carried out in the absence and presence of additives to improve the enzyme’s stability.^32^ Poly(ethylene glycol) (PEG) was used as a stabilizer in different concentrations: 50 mg mL^–1^, 10 mg mL^–1^, 1 mg mL^–1^, 0.5 mg mL^–1^, and 0.05 mg mL^–1^. One milligram of *h*LDH-A was put in contact with 1 g of MCM-41_AG_ in 40 mL of 25 mM carbonate buffer, pH 9 (with PEG in various amounts), and the mixture was kept at 10 °C and gently stirred. The immobilization was monitored as described for the immobilization on MCM-41_A_-GA, vide supra. When the supernatant activity dropped to zero or remained constant for two consecutive samplings, the Schiff bases created between the enzyme and the support were reduced in sodium borohydride solution (0.1 mg mL^–1^ in 100 mL of 25 mM carbonate buffer pH 9 with PEG) for 15 min. Finally, the mixture was filtered under vacuum and washed with 25 mM phosphate buffer, pH 7.5. As previously described, a sample of the filtered solution was collected to monitor whether the phosphate buffer washed away the enzyme. Finally, the imm-*h*LDH-A was washed with distilled water and dried at 4 °C.

##### 2.2.5.3 Immobilization and Immobilized Enzyme Parameters (IY, *R*_act_).

To estimate the immobilization yield (IY), namely, the amount of protein attached on the support surface, the activity of the solution was determined at the beginning (*A*_LDH,*t*_0__) and the end (*A*_LDH,*t*_f__) of the immobilization process, as expressed in eq 5.^32^
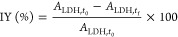
5

The efficiency of imm-*h*LDH-A, obtained by immobilizing the enzyme on the support, was valued according to its retained activity (*R*_act_). *R*_act_ is determined as the ratio between the specific activity of the imm-*h*LDH-A and the specific activity of the free enzyme multiplied by the enzymatic load adopted during the immobilization, as expressed in eq 6:^23^
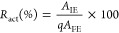
6where *A*_IE_ is the specific activity of the immobilized LDH (U g_supp_^–1^), *q* is the enzymatic load provided during the immobilization (mg_prot_ g_supp_^–1^), and *A*_FE_ is the specific activity of the free LDH (U mg_prot_^–1^), equal to 214 ± 29.2 U mg_prot_^–1^.

#### 2.2.6 Immobilization of Fluorescence-Labeled Enzyme

Optical fluorescence microscopy was performed with the aim of obtaining information about the enzyme presence and distribution on the support. With this purpose, *h*LDH-A was labeled with fluorescence dye (ATTO 488). To label the enzyme, a protein labeling kit and a separation column (purchased from Merck) were used following the procedure provided by the manufacturer; an analogous method was previously employed by Rocha-Martín et al.^33^ The enzyme solution was incubated with ATTO 488 at room temperature in 0.1 M sodium bicarbonate buffer, pH 9.5, for 2 h, the mixture was protected from light and kept under gentle stirring, and finally, it was purified with the separation column. The labeled enzyme was then immobilized according to the immobilization procedure that gave the best results in terms of *R*_act_ and IY, avoiding interaction with light. The imm-*h*LDH-A obtained was observed with a Nikon Eclipse Ti-e fluorescence optical inverted microscope, equipped with a super bright wide-spectrum source (Shutter Lambda XL), a high-resolution camera (Zyla 4.2 Plus, 4098 × 3264 pixels, Andor Technology), and an objective 100× (Nikon) suitable for oil immersion. Brightfield, green, and blue emission images were acquired. The support was observed as a control under the same conditions of analysis.

#### 2.2.7 FT-IR and XRD Characterization of Immobilized *h*LDH-A

A FT-IR spectrum was acquired on imm-*h*LDH-A and compared with the spectrum of the functionalized mesoporous silica obtained under the same conditions. The IR spectra were acquired with a Bruker INVENIO instrument equipped with a liquid nitrogen cooled MCT detector. The samples were degassed to 10^–3^ mbar, and the analyses were performed at room temperature (range 4000–400 cm^–1^, 64 scans, resolution 4 cm^–1^).

In addition, an XRD measurement was carried out on imm-*h*LDH-A with an EMPYREAN diffractometer, Cu Kα radiation, 2θ range of 0.75°–5°, angle step size of 0.013, and 120 s time per step.

#### 2.2.8 Temperature and pH Profile of Free and Immobilized *h*LDH-A

As is known, temperature and pH are two key parameters for the activity and stability of enzymes.^34,35^ Therefore, activity tests were performed on both the free *h*LDH-A and the most active imm-*h*LDH-A (as previously described in section 2.2.1), with varying pH and temperature. To obtain solutions at pH 5 and pH 8, 0.1 M phosphate buffer was used, while 0.1 M carbonate buffer was used for the pH 11 solution. To evaluate the temperature parameter, activity tests were carried out in a temperature range of 25 to 65 °C. The activity is expressed as a percentage of the maximum value obtained.

#### 2.2.9 Thermal Stability of the Free and Immobilized Enzyme

With the purpose of obtaining information concerning the thermal stability of *h*LDH-A in the free and immobilized form, enzymatic assays were performed at different times on the enzymatic solution and the best imm-*h*LDH-A suspension both incubated at pH 7.5 and 45 °C for at least 64 h. For both samples, an incubation temperature of 45 °C was chosen because, from the studies of the pH and temperature profiles, the highest activity for both the free and immobilized enzyme was registered at this temperature vide infra. The activity tests were executed at 35 °C as specified in section 2.2.1.

#### 2.2.10 Cyclic Batch Reaction

With the purpose of obtaining information about the stability of the immobilized enzyme during continuous operations, a cyclic activity test was executed. The imm-*h*LDH-A (0.67 mg mL^–1^) was dispersed in a solution of 1.63 mM pyruvate and 0.23 mM NADH (in 0.1 M phosphate buffer pH 7.5), kept at 30 °C for 60 min. The proceeding of the reaction was followed by using a UV–vis spectrophotometer (Jasco V-730) by monitoring periodically the absorbance at 340 nm and, consequently, by evaluating the concentration of NADH in the solution. After 60 min, the immobilized enzyme was collected by centrifuge and redispersed in a solution with the same concentrations of pyruvate and NADH. The procedure was repeated four times. At the end of each reaction, an activity test on the supernatant was performed to determine whether enzyme leaching occurs.

#### 2.2.11 Inhibition Test with NHI-2

In order to obtain information about the inhibition efficiency of LDH inhibitors on the immobilized enzyme, an imm-*h*LDH-A activity test was performed in the presence of 5.3 μM NHI-2, a well-known LDH inhibitor compound.^36^ The same test was repeated using free *h*LDH-A to evaluate the reliability of the inhibition results obtained with imm-*h*LDH-A. The activity tests were carried out as previously described in the section 2.2.1 adding 20 μL of 0.8 mM NHI-2 diluted in DMSO. The results were presented in terms of activity percentage (%), the activity obtained in the absence of NHI-2 was considered as 100%, and the activity obtained in the presence of the inhibitors was compared to the respective maximum activity. Each test was repeated three times, and results were presented in terms of the average value.

## 3 Results and Discussion

### 3.1 Kinetic Studies of Lactate Dehydrogenase

**Figure 3:**
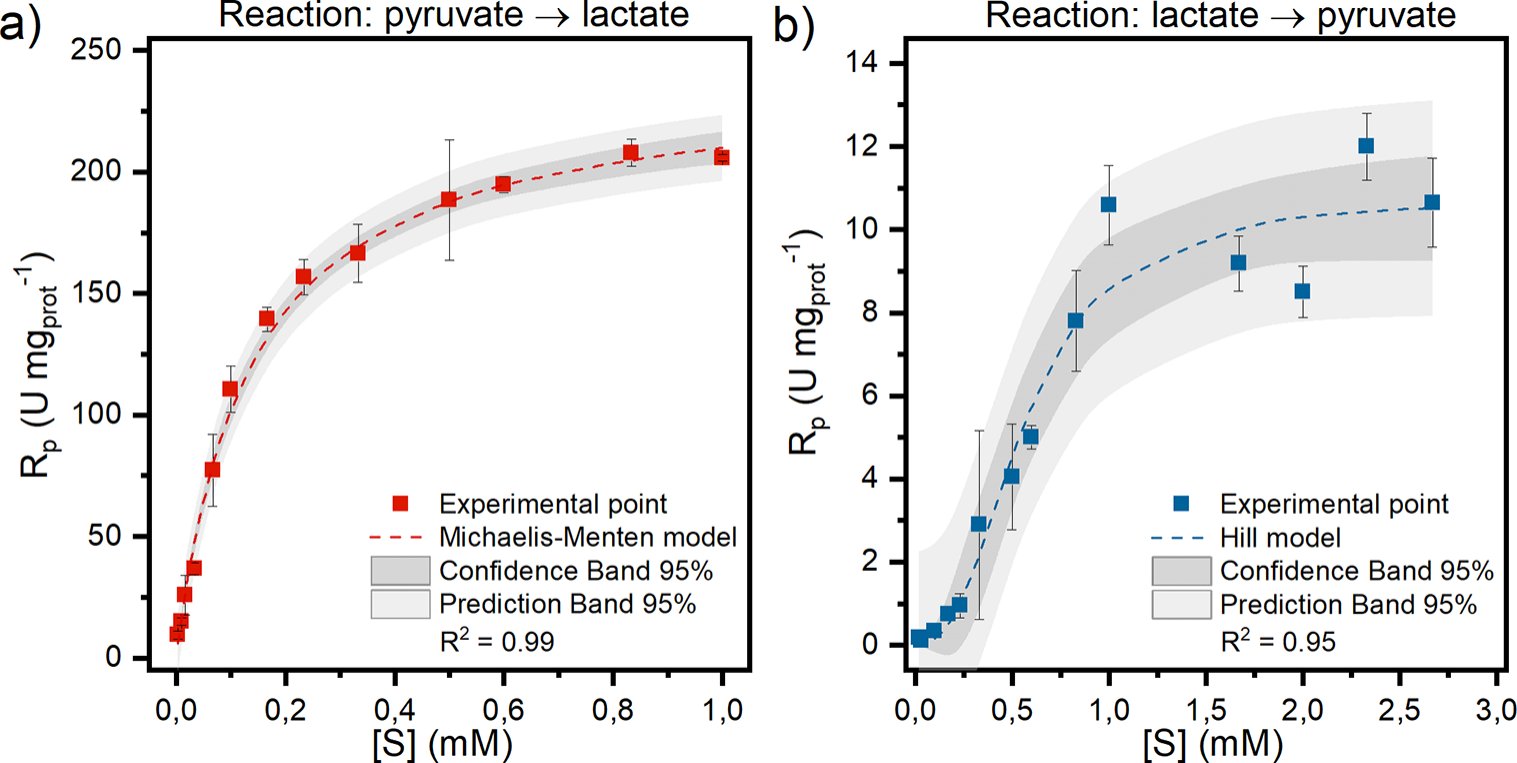
Experimental results achieved for (a) pyruvate reduction and (b) lactate oxidation reactions, along with the corresponding kinetic models. The charts plot the rate of product formation (U mg_prot_^–1^) as a function of the substrate concentration (mmol L^–1^).

The kinetic studies performed on the free enzyme provide information concerning the affinity of *h*LDH-A. As previously described, lactate dehydrogenase is an enzyme consisting of four subunits, which enables it to bind up to four substrates and cofactor molecules.^37^ As with other oligomeric enzymes, lactate dehydrogenase enzyme exhibits allosteric features,^34,37^ namely, its activity is influenced by the binding of ligands that cause a change in its configuration.^38^ When the activity of allosteric enzymes is increased by ligand binding, they demonstrate a cooperative effect.^35^ The kinetic behavior of cooperative enzymes can be properly described by the Hill model, as shown in eq 7.^39^
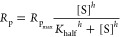
7where *R*_p_ is the rate of product formation (U mg_prot_^–1^), *R*_p_max__ is the maximum rate of product formation (U mg_prot_^–1^), [S] is the substrate concentration (mM), *K*_half_ is the substrate concentration that gives 50% of *R*_p_max__ (mM), and *h* is the adimensional Hill’s coefficient. Consequently, the Michaelis–Menten kinetic model can be described by the Hill equation in the particular case where *h* is equal to 1.^39^ The Michaelis–Menten model is reported in eq 8:
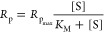
8where *R*_p_ is the rate of product formation (U mg_prot_^–1^), *R*_p_max__ is the maximum rate of product formation (U mg_prot_^–1^), [S] is the substrate concentration (mM), and *K*_M_ is the Michaelis constant (mM). Figure 3 shows the results obtained for both pyruvate reduction (Figure 3a) and lactate oxidation (Figure 3b).

In accordance with findings from Pasti et al.,^40^*h*LDH-A exhibits Michaelis–Menten kinetics when pyruvate reduction is studied at a temperature of 35 °C and pH 7.5, as depicted in Figure 3A. Otherwise, the kinetic behavior of the enzyme approximates a sigmoidal trend described by Hill’s equation. The kinetic parameters for pyruvate reduction are calculated using the Hanes–Woolf linearization method. This approach minimizes experimental errors and results in a better fit of the experimental data compared with the Lineweaver–Burk model, as shown in Figure S3. Conversely, Hill’s parameters (*h* and *K*_half_) for lactate oxidation are estimated using standard regression techniques by analyzing the data graphically. A summary of the kinetic parameters can be found in Table 1.

**Table 1:** Kinetic Parameters (*R*_p_max__, *K*_half_, and Hill’s Coefficient) Estimated for Both Pyruvate Reduction and Lactate Oxidation Reactions

substrate	*R*_p_max__ (U mg_prot_^–1^)	*K*_half_ (mM)	*h*
pyruvate	237.7	0.13	1
lactate	10.7	0.56	2.59

Hill’s coefficient for lactate oxidation is greater than 1, indicating positive cooperativity with respect to lactate binding. Conversely, Hill’s coefficient for pyruvate is equal to 1, indicating no substrate binding cooperativity. In the latter case, it can be assumed that multiple binding sites are present that do not interact cooperatively. As previously mentioned, the initial enzyme concentration is the same for both pyruvate reduction and lactate oxidation tests, but the asymptotic value of *R*_p_ is much higher in the case of pyruvate. The lower value of *K*_half_ for pyruvate reduction suggests a higher affinity of the enzyme for pyruvate than that for lactate as a substrate. These findings are consistent with the earlier statement regarding LDH-A’s affinity for pyruvate.

### 3.2 Support Characterizations

**Figure 4:**
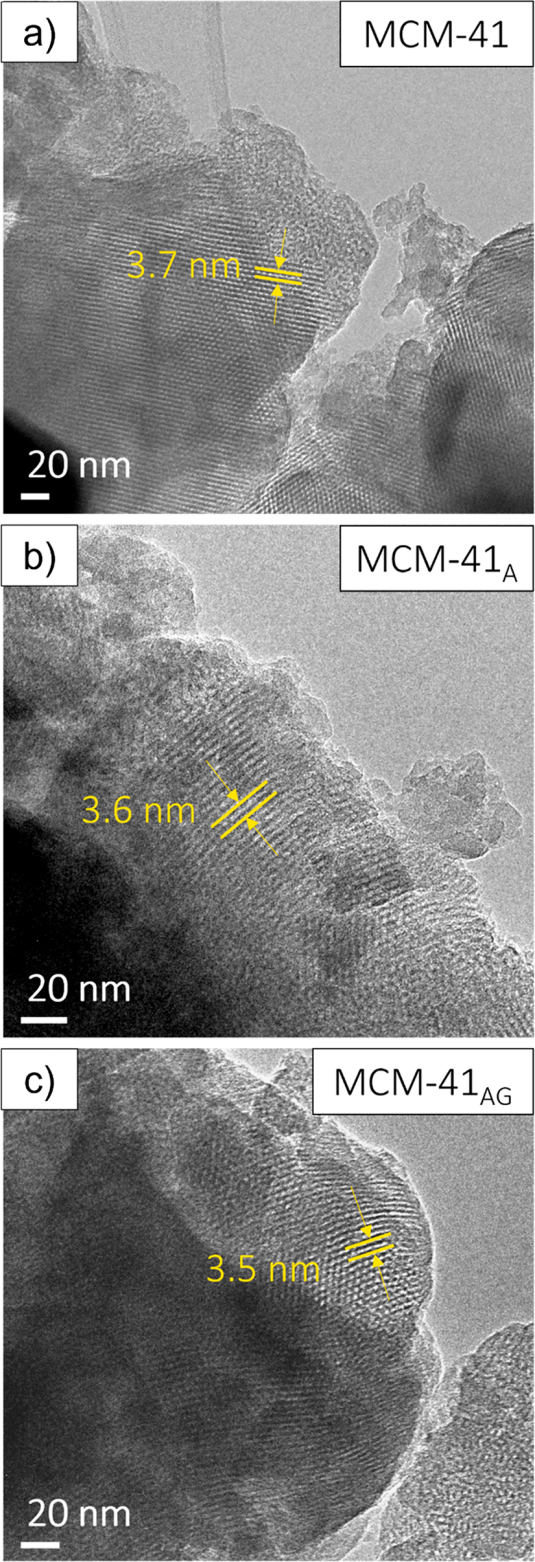
HR-TEM images highlighting the channel distances identified on the MCM-41 (a), MCM-41_A_ (b), and MCM-41_AG_ (c).

The MCM-41 was modified by introducing amino and glyoxyl groups necessary for the immobilization process. The amino groups of MCM-41_A_ (quantified as described in section 2.2.3) are equal to 0.96 mmol g_sup_^–1^. The method used to quantify the amino groups is based on the different concentrations of CuSO_4_ before and after the interaction with MCM-41_A_. As illustrated in Figure S4, CuSO_4_ can be adsorbed on -NH_2_ in different configurations, resulting in an overestimation of the amino group number. The number of glyoxyl groups of MCM-41_AG_ (quantified as described in section 2.2.3) is equal to 1.32 mmol g_sup_^–1^. The structure of the samples was examined through TEM analysis, and the obtained outcomes are shown in Figure 4. No significant changes emerge in MCM-41_A_ (Figure 4b) and MCM-41_AG_ (Figure 4c) in comparison with commercial MCM-41 (Figure 4a). In addition, the channel distances estimated through the Gatan software of TEM reveal no inner transformation in the materials. It can be concluded that the functionalization process does not modify the morphology of the mesoporous silica.

**Figure 5:**
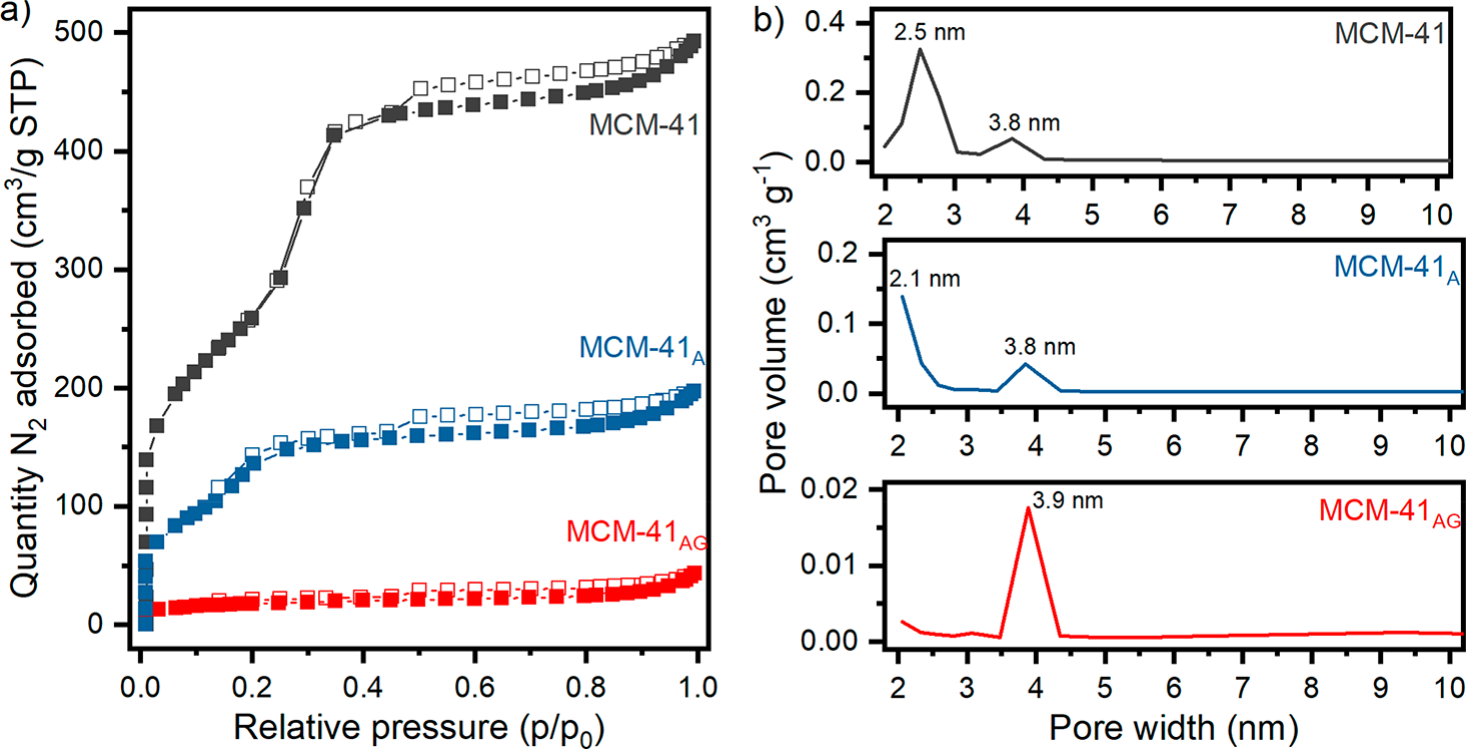
(a) N_2_ physisorption isotherms at −196 °C of pristine and functionalized MCM-41. (b) Pore size distributions of MCM-41, MCM-41_A_, and MCM-41_AG_. All the samples were pretreated at 200 °C for 2 h.

The unmodified (MCM-41) and functionalized (MCM-41_A_, MCM-41_AG_) silica were characterized through N_2_ physisorption at −196 °C, as shown in Figure 5. MCM-41 displays a type IV adsorption isotherm (Figure 5a), according to the IUPAC classification, characteristic of mesoporous materials.^41^

On the other hand, MCM-41_AG_ shows a type II adsorption isotherm and MCM-41_A_ presents a combination of type II and type IV adsorption isotherms.

As a result of the functionalization process, the specific surface area decreases by ∼53% for MCM-41_A_ and ∼95% for MCM-41_AG_, as does the total pore volume. These effects may be due to the occlusion of the smaller pores by APTES and GPTMS.^42,43^ As can be seen in Figure 5b, MCM-41 presents pores of two sizes; after the functionalization the minor pores are partially reduced by the presence of organosilanes (MCM-41_A_), while for MCM-41_AG_ the pores around 2 nm disappeared, in agreement with *d*_BJH_ reported in Table 2.

**Table 2:** Results of Nitrogen Physisorption at −196 °C of MCM-41, MCM-41_A_, and MCM-41_AG_

	*S*_BET_^a^ (m^2^ g^–1^)	*V*_p_^b^ (cm^3^ g^–1^)	*d*_BJH_^c^ (nm)
MCM-41	1085	0.74	2.7
MCM-41_A_	509	0.29	2.3
MCM-41_AG_	58	0.06	3.8

a Specific surface area calculated through the BET method.

b Total pore volume calculated through the BJH method.

c Mean pore diameter calculated through the BJH method.

Table 2 summarizes the results of N_2_ physisorption at −196 °C.

The minimum diameter of the enzyme can be estimated by using eq 9, where *D*_min_ is the minimum diameter of the enzyme whose shape is approximated to a sphere (nm) and *M* is the molecular weight of the enzyme (Da).^26^
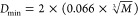
9

The molecular weight of *h*LDH-A is reported to be 27.46 kDa (Uniprot ID. K1T0A2). Consequently, the minimum diameter of *h*LDH-A is 3.98 nm; therefore its size is comparable to the largest pores of the supports, i.e., 3.8 nm as obtained for the MCM-41_AG_ from Table 2 and Figure 5b.

**Figure 6:**
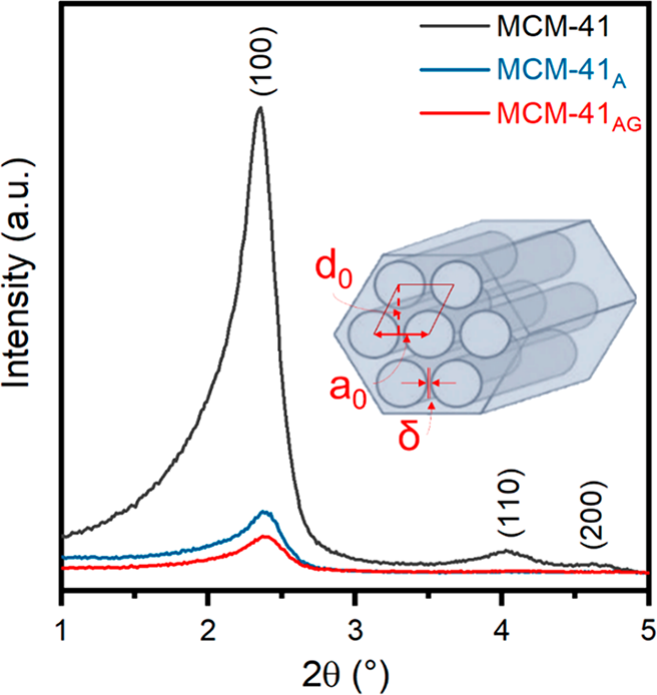
X-ray diffractograms of MCM-41, MCM-41_A_, and MCM-41_AG_ at low angles (2θ range 1°–5°) and scheme of the MCM-41 cell parameters: inter-reticular distance (*d*_0_), cell parameter (*a*_0_), and wall thickness (δ).

Figure 6 shows the XRD patterns. As a whole, the unmodified silica presents three peaks in the 2θ range 1°–5° ascribed to (100) at 2θ = 2.36°, (110) at 2θ = 4.03°, and (200) at 2θ = 4.62° characteristic of MCM-41.^42^

As previously reported in the literature, the diffractograms of MCM-41_A_ and MCM-41_AG_ look like those typical of amorphous materials and present (100) peaks of lower intensity and slightly shifted to higher 2θ values (2.37° and 2.38° for MCM-41_A_ and MCM-41_AG_, respectively). These results can be attributed to the organic groups of APTES and GPTMS attached to the mesoporous silica surface which reduce the scattering intensities of the material.^43−45^

**Figure 7:**
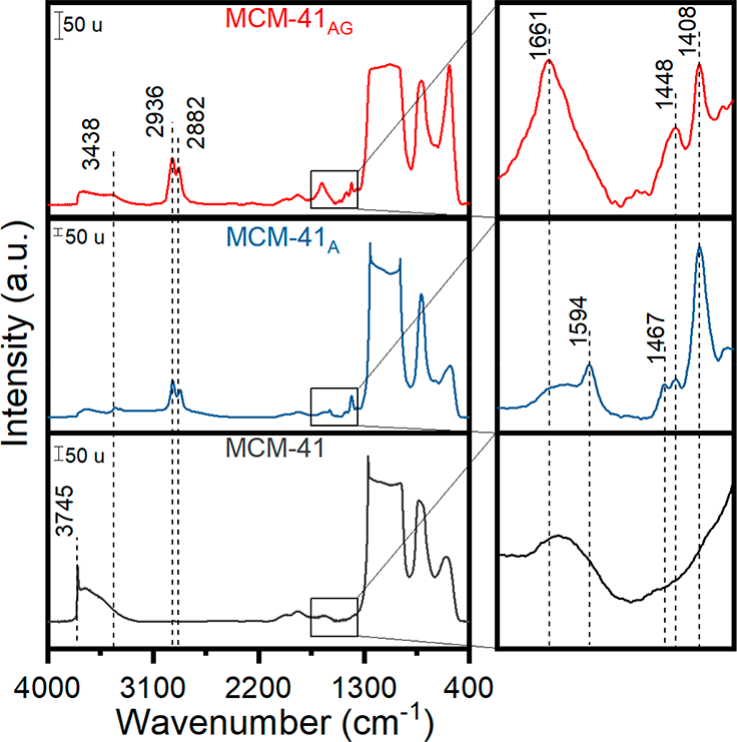
FT-IR spectra of MCM-41, MCM-41_A_, and MCM-41_AG_. On the left is shown the magnification of the range of 1750–1350 cm^–1^. The spectra were acquired at room temperature after being pretreated at 300 °C in standard vacuum conditions for 1 h.

To confirm the successful functionalization of the materials, the functionalized samples were analyzed using FT-IR and compared to the unmodified MCM-41 (Figure 7).

As a whole, MCM-41_A_ and MCM-41_AG_ present a peak at 3428 cm^–1^ due to -NH stretching^46^ and two signals at 2936 and 2882 cm^–1^ ascribed to symmetric and asymmetric stretching of −CH_2_ present in GPTMS and APTES.^47,48^ At lower wavenumbers, the band centered at 1661 cm^–1^ can be assigned to the carbonyl group signal,^46^ whereas peaks at 1594 and 1448 cm^–1^ are attributable to -NH_2_ deformation vibrations.^49^ The signal at 1467 cm^–1^ can be ascribed to −CH_2_ bending vibration,^46^ and the peak at 1408 cm^–1^ can be ascribed to -Si-CH_2_ deformation.^47^ On the other hand, the commercial MCM-41 presents an intense peak at 3745 cm^–1^ assigned to isolated silanols.^50^ This last peak is not present in the spectra of MCM-41_A_ and MCM-41_AG_ due to the functionalization process during which organosilanes react with the SiOH groups.

### 3.3 *h*LDH-A Immobilization on MCM-41_A_ Preactivated with Glutaraldehyde and MCM-41_AG_

The enzyme was immobilized on MCM-41_A_ and MCM-41_AG_ following the two different procedures described in section 2.2.5.

*h*LDH-A was immobilized on MCM-41_A_ activated with 2.5% v/v and 1% v/v glutaraldehyde, a bifunctional cross-linking agent reactive with lysine residues of the enzyme.^20,30,31^ A two-step immobilization procedure was adopted to prevent the deactivation of *h*LDH-A due to inter- and intra-cross-linking by glutaraldehyde, a molecule sufficiently small to reach the active site of *h*LDH-A and react with the groups involved in the catalytic process.^51^ In the first step the support was activated separately from *h*LDH-A; subsequently, MCM-41_A_-GA was introduced in the enzymatic solution.

Otherwise, multipoint immobilization of *h*LDH-A was performed by putting it in contact with the heterofunctional mesoporous silica (MCM-41_AG_) in a carbonate buffer at pH 9. The alkaline pH is required to deprotonate the lysine residues and make them react with the support glyoxyl groups. The immobilization was carried out at pH 9 which provides the alkaline environment necessary to obtain the enzyme immobilization (it is usually made at pH 10) and avoid the denaturation of the protein.^20,32^ The isoelectric point of lactate dehydrogenase is around pH 8; at higher pH the enzyme is negatively charged.^51^ The immobilization was performed in the presence of PEG in different concentrations, used as a stabilizer to protect the enzyme from the alkaline environment. The same immobilization was conducted without the stabilizer to evaluate the role of PEG on the activity of the imm-*h*LDH-A. Table 3 summarizes the parameters obtained by the two methods. In all of the conditions, no enzyme was detected in the filtered solution after washing.

**Table 3:** Immobilizations Conditions Tested and Activities Obtained for Immobilized *h*LDH-A

support	immobilization condition	IY (%)	activity (U)	*A*_IE_ (U g^–1^)	*Q* (mg_prot_ g_sup_^–1^)	*R*_act_ (%)
MCM-41_A_–GA-1^a^	no stabilizer	95.7	0.11 ± 0.04	55.9 ± 21.5	2	13.1
MCM-41_A_–GA-2.5^a^	no stabilizer	94.8	0.07 ± 0.01	37.1 ± 5.1	2	8.7
MCM-41_AG_	no stabilizer	99.1	0.06 ± 0.03	28.9 ± 14.5	1	13.5
	PEG 50 mg/mL	79.6	0.03 ± 0.02	15.1 ± 7.9	1	7.0
	PEG 10 mg/mL	79.8	0.04 ± 0.02	21.7 ± 7.6	1	10.1
	PEG 1 mg/mL	100	0.08 ± 0.03	39.1 ± 14.7	1	18.3
	PEG 0.5 mg/mL	95.9	0.05 ± 0.03	26.5 ± 14.6	1	12.4
	PEG 0.05 mg/mL	91.1	0.10 ± 0.01	51.8 ± 5.9	1	24.2

a Concentration of glutaraldehyde in solution during the preactivation step, namely, 1% v/v and 2.5% v/v.

All the immobilizations resulted in immobilization yields (IY) higher than 79%, with the majority of them exceeding 90%. Despite the high values of immobilization yields, the retained activities (*R*_act_) range from 7% to 24.2%, with most values close to 10%.

As can be seen, by increasing the glutaraldehyde concentration of the preactivation step of MCM-41_A_, the *R*_act_ decreases. This effect may be due to the stiffening of the protein resulting from the excessive binding between the enzyme and the support.^18^ Unexpectedly, for the multipoint covalent immobilization, increasing the stabilizer concentration seems to worsen the retained activity, while at a low concentration, the presence of PEG improves *R*_act_. A possible explanation for this phenomenon is that PEG, when present in concentrations exceeding 10 mg/mL, obstructs the interaction between the enzyme and the support. Consequently, the immobilizations carried out with 50 and 10 mg/mL have demonstrated the lowest immobilization yields. On the other hand, concentrations of 1 or 0.5 mg/mL of PEG do not hinder the interaction of the protein with the functional groups of the support, but it can interfere with the hydrophobic pockets of the enzyme changing its structure, as previously reported in the presence of glycerol by Braham et al.^52^ In particular, the positive effect of stabilizing obtained with a concentration of 1 mg/mL of PEG may be greater than the negative phenomenon of enzymatic pocket obstruction. The best result in terms of *R*_act_ was obtained through the multipoint covalent immobilization over MCM-41_AG_ in the presence of 0.05 mg/mL of PEG. Therefore, the following characterizations of the imm-*h*LDH-A were carried out using these latter immobilization conditions.

### 3.4 Characterizations of the Immobilized Lactate Dehydrogenase

**Figure 8:**
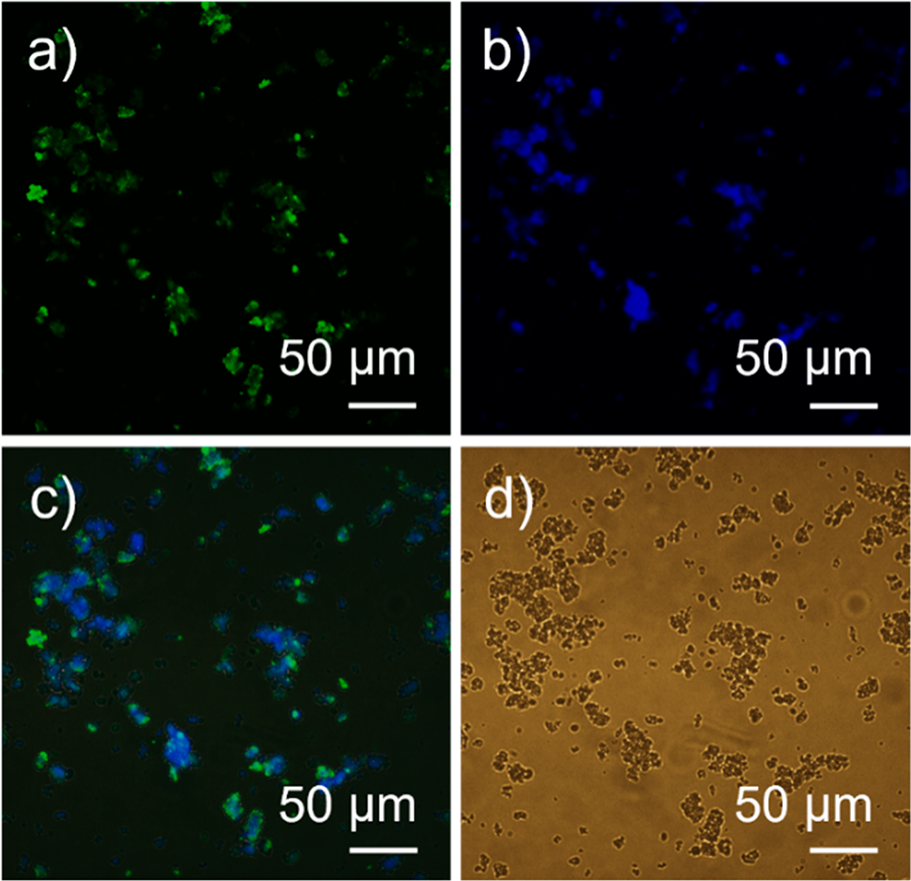
Optical fluorescence microscopy images of *h*LDH-A labeled with ATTO 488 immobilized on MCM-41_AG_ in the green channel (a), blue channel (b), green and blue channels overlapped (c), and brightfield (d). The brightness and contrast of the images were corrected.

To verify the presence and distribution of the enzyme immobilized on MCM-41_AG_, optical fluorescence microscopy was conducted. The resulting images of the imm-*h*LDH-A were collected in the green emission channel (Figure 8a), the blue emission channel (Figure 8b), and brightfield (Figure 8d), as shown in Figure 8. Additionally, the support alone was analyzed with optical fluorescence microscopy to assess the intensity of its intrinsic fluorescence emission (Figure S5).

The enzyme can be easily distinguished in Figure 8a,c as spots of very intense green emissions. The blue spots appearing in Figure 8b,c allow the localization of MCM-41_AG_ particles in the images. The images obtained by fluorescence microscopy confirm the presence of the enzyme on the silica surface, verifying the success of the immobilization (Figure 8c). While it is obviously not possible to distinguish individual enzymatic macromolecules due to the limitations of optical microscopy, the enzymatic spots appear well-distributed in the image, suggesting a macroscopically homogeneous immobilization (Figure 8a). The support itself, observed as a control, has a negligible emission in the green channel and a slight fluorescence emission in the blue channel (Figure S5), which increases when the concentration of particles is high. The blue emission fluorescence of MCM-41_AG_ can be a side effect of the functionalization process, which is carried out in a toluene solution and provides −COH and amino groups.^29^ The images obtained by fluorescence microscopy confirmed the presence of the enzyme on the silica surface and, therefore, the fact that immobilization has occurred (Figure 8c).

**Figure 9:**
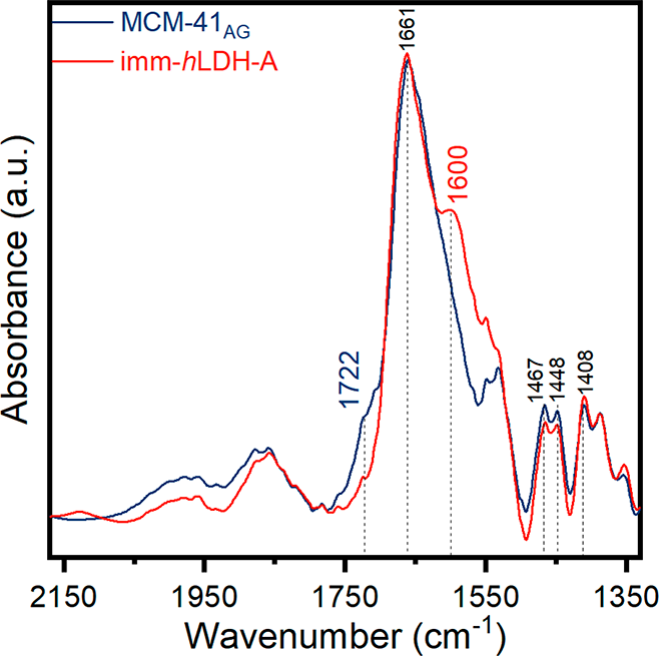
MCM-41_AG_ and imm-*h*LDH-A FT-IR spectra collected at 10^–3^ mbar in the range 1350–2150 cm^–1^.

In order to obtain information about the covalent bond established between *h*LDH-A and MCM-41_AG_ and further evidence of the presence of the enzyme on mesoporous silica, FT-IR and XRD measurements were acquired on imm-*h*LDH-A. Figure 9 shows the IR spectra collected on imm-*h*LDH-A and MCM-41_AG_. The imm-*h*LDH-A spectrum displays a peak at 1600 cm^–1^, which can be correlated to the stretching signal of amide I, whose general formula is R-C(=O)-NR′R″.^53,54^ Moreover, the MCM-41_AG_ spectrum presents a shoulder at 1722 cm^–1^ that can be correlated to −CH_2_–C=O stretching signal provided by the third functionalization step (Figure S1 for further details).^49^ The absence of the carbonyl group signal at 1722 cm^–1^ in the imm-*h*LDH-A spectrum can suggest that C=O support groups are involved in the covalent bonds between carbonyl groups of the support and the -NH_2_ groups of the enzyme (as schematized in Figure S2b).

Concerning the XRD diffractogram of imm-*h*LDH-A (Figure S6), new peaks related to the enzyme are not observed.^55^ The intensity of the (100) peak is further decreased by the presence of the enzyme^56^ and shifted to a higher 2θ value. This phenomenon can be related to the occlusion of MCM-41 pores by the organic layer of *h*LDH-A, which amplifies what was previously observed after the functionalization with APTES and GPTMS, previously described for MCM-41_A_ and MCM-41_AG_ diffractograms (section 3.2).

The enzymatic activity is greatly affected by the temperature and pH, highlighting the importance of operating under appropriate conditions for both factors.^34^ To gather insights into the activity of *h*LDH-A in both free and immobilized configurations under different pH and temperature values, multiple tests were conducted by varying these experimental parameters. These tests aimed to identify the environment that would result in maximum enzyme activity.

**Figure 10:**
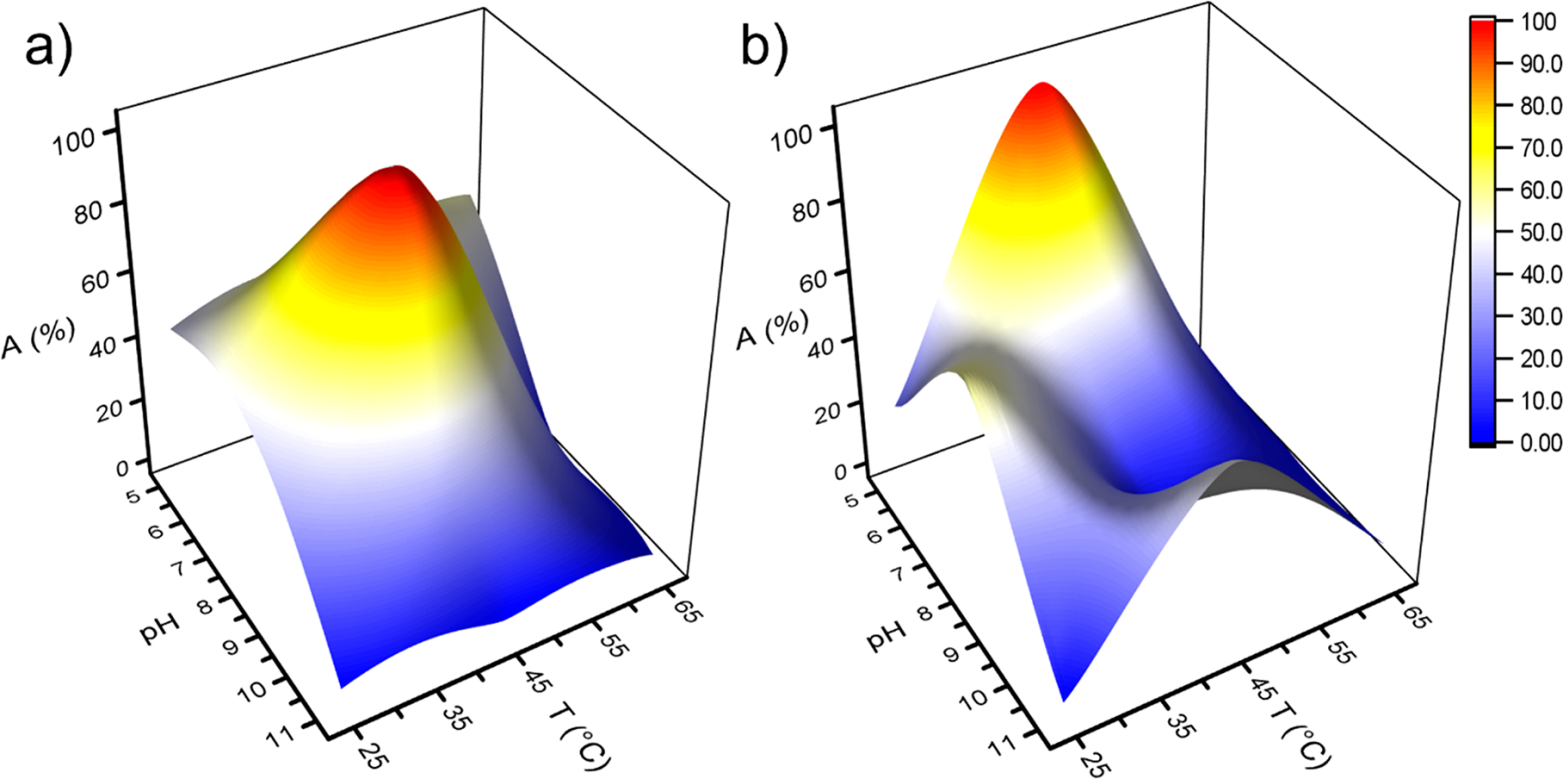
Activity (%) map as a function of pH and temperature for free (a) and immobilized *h*LDH-A (b).

The tested pH and temperature values are listed in Figure S7, while the graphical representation of the results and the corresponding activity (%) values are shown in Figure 10 and Table S1, respectively.

The free enzyme exhibits the highest activity at pH 8 and 45 °C, while the immobilized enzyme has its maximum activity at pH 5 and 45 °C. The activity of the free enzyme is more affected by the pH than by the temperature, as evidenced by the lowest activities recorded at pH 11. In contrast, the activity of the immobilized *h*LDH-A exhibits a fluctuating trend with an absolute value (pH 5 and *T* 45 °C) and two relative maximum values (pH 8 and *T* 25 °C; pH 11 and *T* 45 °C). Previous studies have reported that mesoporous silica surfaces can buffer the surrounding environment, resulting in a less acidic surrounding than the bulk solution.^57^ It is therefore reasonable to suppose that although the test was performed at pH 5, the environment of the enzyme was closer to a neutral pH value. At the extreme boundaries of the pH range (i.e., at pH 5 and 11), the imm-*h*LDH-A’s activity seems to follow the same trend as the free enzyme at the temperature variations, and its activity seems to be favored by a temperature of 45 °C. However, at lower temperatures, slightly alkaline conditions (pH 8) promote imm-*h*LDH-A’s activity.

**Figure 11:**
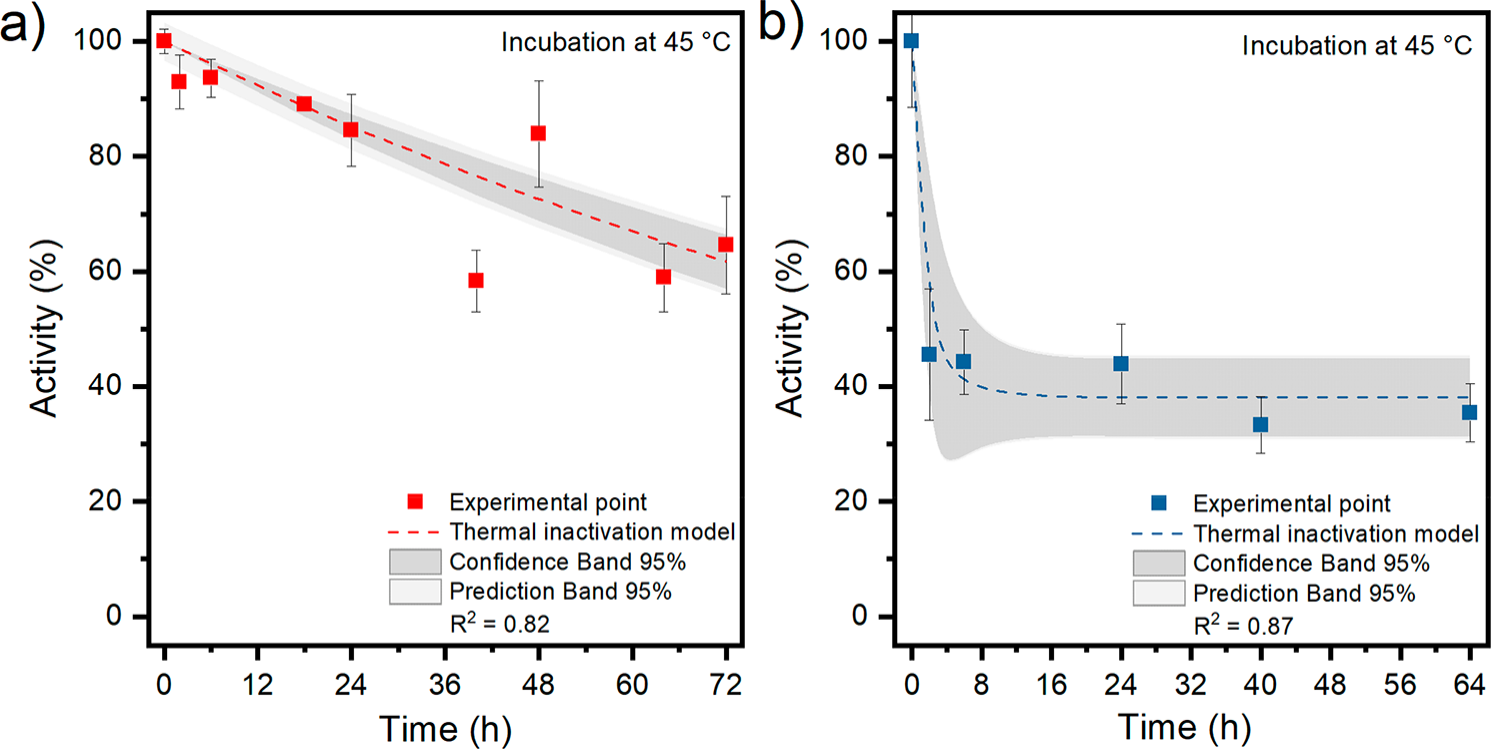
Thermal inactivation evaluated for both free (a) and immobilized (b) lactate dehydrogenase. Both the enzymatic solution and the biocatalyst suspension were incubated at pH 7.5 and 45 °C. The activity tests were performed at 35 °C.

To investigate the thermal deactivation of lactate dehydrogenase in both its free and immobilized conditions, the *h*LDH-A solution and imm-*h*LDH-A suspension were incubated at 45 °C (the temperature at which the highest activity was registered for both forms) for at least 64 h before being tested at 35 °C. The results of these tests, presented as a percentage of the initial activity, are shown in Figure 11.

Both free *h*LDH-A and the imm-*h*LDH-A display an exponential inactivation curve, expressed by eqs 10 and 11, respectively:
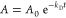
10
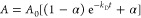
11where *A* is the activity, *A*_0_ is the initial activity, α is the residual activity at infinite time, *k*_D_ is the deactivation constant (h^–1^), and *t* is the time (h). The models reported were previously described by Illanes et al.^58^ The residual activity (α), the inactivation constant (*K*_D_), and the half-life (*t*_1/2_) are summarized in Table 4.

**Table 4:** Thermal Inactivation Results for Free and Immobilized Lactate Dehydrogenase Obtained after Incubation at 45 °C

	*K*_D_ (h^–1^)	α (%)	*t*_1/2_ (h)
free *h*LDH-A	0.007		103
immobilized *h*LDH-A	0.962	38	2

The initial slope of the curve interpolating the experimental data is greater for the immobilized enzyme than the free one. This outcome is in accordance with the obtained values of the inactivation constant, because a higher K_D_ implies a faster deactivation of the enzyme. Despite the lower half-life, the immobilized *h*LDH-A maintains a residual activity (α) of 38% for an extended period of time, whereas the free enzyme has a half-life of 103 h and will eventually lose all its activity. In the perspective of the realization of a biosensor, imm-*h*LDH-A should have high long-term stability to perform reliable comparison among the different *h*LDH-A inhibitor compounds.

Further research should be undertaken to investigate how the different immobilization conditions (e.g., the amount of functional groups on the surface of support, concentration and type of stabilizer during the immobilization procedure) affect the activity and the long-term stability of the imm-*h*LDH-A obtained.

### 3.5 Preliminary Tests of Biosensor Feasibility

**Figure 12:**
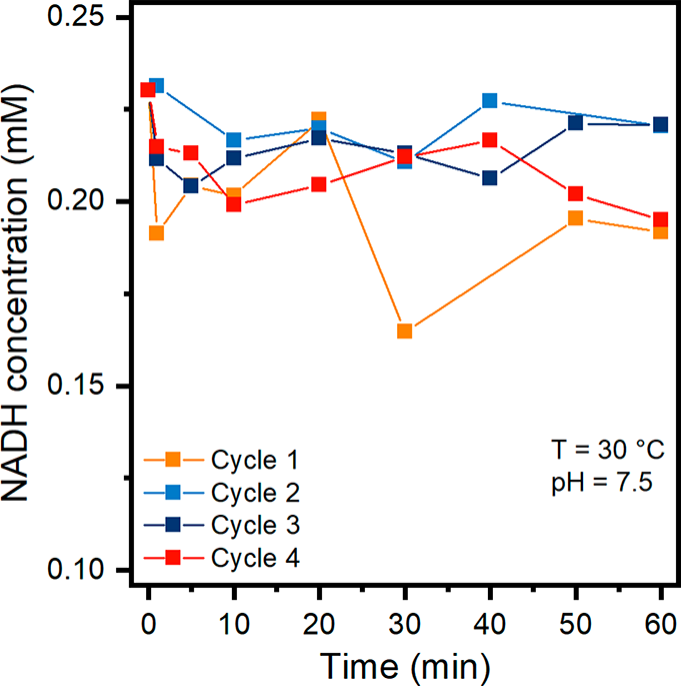
NADH concentrations during the cyclic test comprising four consecutive cycles.

In order to study the stability of the immobilized enzyme in continuous operations, imm-*h*LDH-A was subjected to a cyclic test comprising four consecutive cycles, each lasting 60 min (Figure 12).

After an initial decrease, the NADH concentration fluctuates. This phenomenon is attributed to an equilibrium condition with the reverse reaction during which NADH is oxidized to NAD^+^.^26^ The activity of imm-*h*LDH-A remains quite stable during the four cycles, and the NADH concentration measured at the end of the fourth cycle (195 ± 6 μM) is very similar to the concentration at the end of the first cycle (192 ± 9 μM). No activity was detected in the supernatant at the end of each cycle. Therefore, it can be supposed that enzyme leaching does not occur. This test may suggest that reuse of the imm-*h*LDH-A is feasible; by contrast, the free enzyme could not be recovered at the end of the reaction.

**Figure 13:**
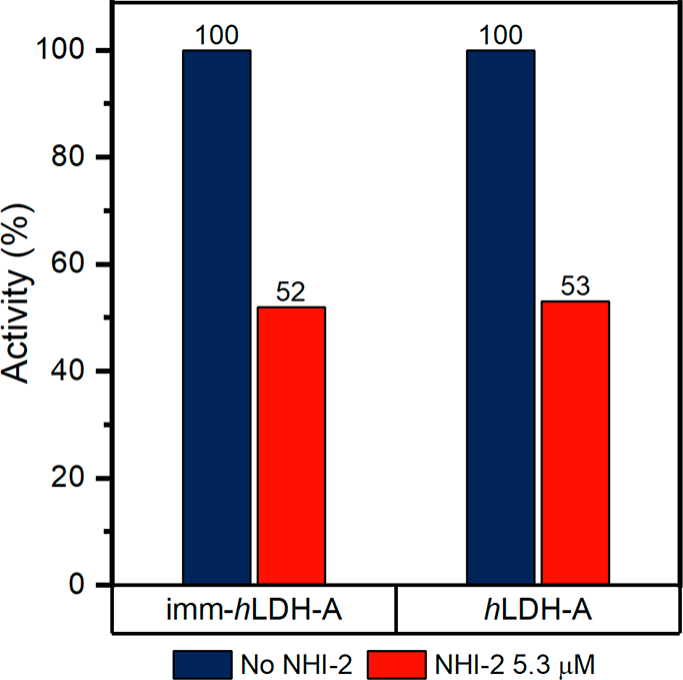
Activity (%) of *h*LDH-A and imm-*h*LDH-A in the presence and absence of NHI-2.

With the purpose of obtaining information about the effective employment of imm-*h*LDH-A as a catalyst used in a biosensor for the screening of *h*LDH-A drug inhibitors, an activity test in the presence of 5.3 μM NHI-2 was performed on both free *h*LDH-A and imm-*h*LDH-A. Figure 13 displays the outcomes obtained.

The activity inhibition of imm-*h*LDH-A caused by the presence of NHI-2 is 48%, and a similar result is obtained for the free *h*LDH-A whose activity is inhibited by 47% by the presence of the drug. Therefore, it can be assumed that the inhibition efficiency of NHI-2 is not affected by the fact that the enzyme is in its immobilized or free form.

These outcomes suggest that the realization of a biosensor based on imm-*h*LDH-A for the screening of LDH-A inhibitors is possible.

## 4 Conclusions

In this study, the lactate dehydrogenase enzyme (*h*LDH-A, EC 1.1.1.27) from humans, expressed in *E. coli*, was covalently immobilized on functionalized MCM-41. The results of support characterization analyses confirmed the effective functionalization of mesoporous silica. Two immobilization procedures were employed for the enzyme immobilization, and different immobilization conditions were tested for each procedure. The best results in terms of retained activity (*R*_act_ = 24.2%) were obtained by immobilizing *h*LDH-A on MCM-41 functionalized with amino and glyoxyl groups in the presence of a concentration equal to 0.05 mg/mL PEG. This immobilization was also repeated with a fluorescent-dye-labeled enzyme, and the resulting imm-*h*LDH-A was observed using optical fluorescence microscopy. The acquired images confirmed the presence of the enzyme on MCM-41. The imm-*h*LDH-A was characterized to study its activity as a function of pH and temperature as well as its thermal stability. The outcomes of the imm-*h*LDH-A characterization analyses were compared with those of the free enzyme: the highest activity of the best imm-*h*LDH-A was obtained at pH 5 and 45 °C for the reduction of pyruvate into lactate, while the free enzyme showed its highest activity at pH 8 and 45 °C. Concerning the thermal stability, despite a low half-life time, imm-*h*LDH-A has a residual activity equal to 38%. The activity test performed in the presence of NHI-2 reveals a similar inhibition both for the free (47%) and for the immobilized *h*LDH-A (48%). These findings provide insights into the development of a biosensor based on immobilized lactate dehydrogenase. The results obtained are also of interest to those who wish to immobilize multimeric enzymes on mesoporous silica. The retained activity obtained in this study was 24.2%, which indicates that future work should focus on further optimizing the immobilization procedure to enhance the activity and stability of the biocatalyst. Furthermore, customized mesoporous silica can be ad-hoc synthesized and used as a support for this enzyme, because the appropriate support can improve the biocatalyst’s properties.

## Supporting Information Available

Scheme of the functionalization process performed on MCM-41 to provide amino and aldehyde (glyoxyl) functional groups, detailed scheme of the immobilization process of *h*LDH-A on MCM-41_A_, preactivated with glutaraldehyde, and MCM-41_AG_, comparison between Lineweaver–Burk and Hanes–Woolf linearization models applied to the results of the kinetic tests, evaluation of the amino groups present on the surface of the MCM-41 after the functionalization process, description of the procedure used for the evaluation of MCM-41 cell parameters, optical fluorescence microscopy image acquired on MCM-41_AG_, X-ray diffractograms of MCM-41, MCM-41_A_, MCM-41_AG_, and imm-hLDH-A at low angles (2θ range 2°–5°), map of the pH and temperature conditions analyzed, activity (%) results obtained as a function of pH and temperature (°C) for free and immobilized *h*LDH-A (PDF)

## References

[ref1] Sung H.; Ferlay J.; Siegel R. L.; Laversanne M.; Soerjomataram I.; Jemal A.; Bray F. Global Cancer Statistics 2020: GLOBOCAN Estimates of Incidence and Mortality Worldwide for 36 Cancers in 185 Countries. CA. Cancer J. Clin. 2021, 71 (3), 209–249. 10.3322/caac.21660. 33538338

[ref2] Global Cancer Observatory https://gco.iarc.fr/.

[ref3] Gan J.; Wang W.; Yang Z.; Pan J.; Zheng L.; Yin L. Prognostic Value of Pretreatment Serum Lactate Dehydrogenase Level in Pancreatic Cancer Patients A Meta-Analysis of 18 Observational Studies. Medicine (United States) 2018, 97, e13151 10.1097/MD.0000000000013151. PMC6257391 30431587

[ref4] Varma G.; Seth P.; Coutinho de Souza P.; Callahan C.; Pinto J.; Vaidya M.; Sonzogni O.; Sukhatme V.; Wulf G. M.; Grant A. K. Visualizing the Effects of Lactate Dehydrogenase (LDH) Inhibition and LDH-A Genetic Ablation in Breast and Lung Cancer with Hyperpolarized Pyruvate NMR. NMR Biomed. 2021, 34 (8), e4560 10.1002/nbm.4560. 34086382 PMC8764798

[ref5] Wei Y.; Xu H.; Dai J.; Peng J.; Wang W.; Xia L.; Zhou F. Prognostic Significance of Serum Lactic Acid, Lactate Dehydrogenase, and Albumin Levels in Patients with Metastatic Colorectal Cancer. Biomed Res. Int. 2018, 2018, 1804086 10.1155/2018/1804086. 30627541 PMC6304480

[ref6] Cui B.; Luo Y.; Tian P.; Peng F.; Lu J.; Yang Y.; Su Q.; Liu B.; Yu J.; Luo X.; Yin L.; Cheng W.; An F.; He B.; Liang D.; Wu S.; Chu P.; Song L.; Liu X.; Luo H.; Xu J.; Pan Y.; Wang Y.; Li D.; Huang P.; Yang Q.; Zhang L.; Zhou B. P.; Liu S.; Xu G.; Lam E. W. F.; Kelley K. W.; Liu Q. Stress-Induced Epinephrine Enhances Lactate Dehydrogenase A and Promotes Breast Cancer Stem-like Cells. J. Clin. Invest. 2019, 129 (3), 1030–1046. 10.1172/JCI121685. 30688660 PMC6391112

[ref7] Sun L.; Li J.; Yan W.; Yao Z.; Wang R.; Zhou X.; Wu H.; Zhang G.; Shi T.; Chen W. H19 Promotes Aerobic Glycolysis, Proliferation, and Immune Escape of Gastric Cancer Cells through the MicroRNA-519d-3p/Lactate Dehydrogenase A Axis. Cancer Sci. 2021, 112 (6), 2245–2259. 10.1111/cas.14896. 33756038 PMC8177792

[ref8] Cascardo F.; Anselmino N.; Páez A.; Labanca E.; Sanchis P.; Antico-Arciuch V.; Navone N.; Gueron G.; Vázquez E.; Cotignola J. Ho-1 Modulates Aerobic Glycolysis through Ldh in Prostate Cancer Cells. Antioxidants 2021, 10 (6), 966 10.3390/antiox10060966. 34208670 PMC8235201

[ref9] Zhang S. L.; He Y.; Tam K. Y. Targeting Cancer Metabolism to Develop Human Lactate Dehydrogenase (HLDH)5 Inhibitors. Drug Discovery Today 2018, 23, 1407–1415. 10.1016/j.drudis.2018.05.014. 29750903

[ref10] Kobayashi Y.; Banno K.; Kunitomi H.; Takahashi T.; Takeda T.; Nakamura K.; Tsuji K.; Tominaga E.; Aoki D. Warburg Effect in Gynecologic Cancers. J. Obstet. Gynaecol. Res. 2019, 45 (3), 542–548. 10.1111/jog.13867. 30511455

[ref11] Ippolito L.; Morandi A.; Giannoni E.; Chiarugi P. Lactate: A Metabolic Driver in the Tumour Landscape. Trends Biochem. Sci. 2019, 44 (2), 153–166. 10.1016/j.tibs.2018.10.011. 30473428

[ref12] Mishra D.; Banerjee D. Lactate Dehydrogenases as Metabolic Links between Tumor and Stroma in the Tumor Microenvironment. Cancers (Basel). 2019, 11 (6), 750 10.3390/cancers11060750. 31146503 PMC6627402

[ref13] de la Cruz-López K. G.; Castro-Muñoz L. J.; Reyes-Hernández D. O.; García-Carrancá A.; Manzo-Merino J. Lactate in the Regulation of Tumor Microenvironment and Therapeutic Approaches. Front. Oncol. 2019, 9, 1143 10.3389/fonc.2019.01143. 31737570 PMC6839026

[ref14] Kusumawati R.; Nasrullah A. H.; Pesik R. N.; Muthmainah; Indarto D. Secondary Metabolites of Mirabilis Jalapa Structurally Inhibit Lactate Dehydrogenase A in Silico: A Potential Cancer Treatment. In IOP Conference Series: Materials Science and Engineering; Institute of Physics Publishing, 2018; Vol. 333, 10.1088/1757-899X/333/1/012078.

[ref15] Augoff K.; Hryniewicz-Jankowska A.; Tabola R. Lactate Dehydrogenase 5: An Old Friend and a New Hope in the War on Cancer. Cancer Letters. 2015, 358, 1–7. 10.1016/j.canlet.2014.12.035. 25528630

[ref16] Cheng G.; Pi Z.; Zheng Z.; Liu S.; Liu Z.; Song F. Magnetic Nanoparticles-Based Lactate Dehydrogenase Microreactor as a Drug Discovery Tool for Rapid Screening Inhibitors from Natural Products. Talanta 2020, 209, 120554 10.1016/j.talanta.2019.120554. 31892010

[ref17] Zhou Y.; Tao P.; Wang M.; Xu P.; Lu W.; Lei P.; You Q. Development of Novel Human Lactate Dehydrogenase A Inhibitors: High-Throughput Screening, Synthesis, and Biological Evaluations. Eur. J. Med. Chem. 2019, 177, 105–115. 10.1016/j.ejmech.2019.05.033. 31129449

[ref18] Barbosa O.; Torres R.; Ortiz C.; Berenguer-Murcia Á.; Rodrigues R. C.; Fernandez-Lafuente R. Heterofunctional Supports in Enzyme Immobilization: From Traditional Immobilization Protocols to Opportunities in Tuning Enzyme Properties. Biomacromolecules 2013, 14, 2433–2462. 10.1021/bm400762h. 23822160

[ref19] Arana-Peña S.; Carballares D.; Morellon-Sterlling R.; Berenguer-Murcia Á.; Alcántara A. R.; Rodrigues R. C.; Fernandez-Lafuente R. Enzyme Co-Immobilization: Always the Biocatalyst Designers’ Choice. . .or Not?. Biotechnology Advances 2021, 51, 107584 10.1016/j.biotechadv.2020.107584. 32668324

[ref20] Immobilization of Enzymes and Cells, 3rd ed.; Guisan J. M., Ed.; Humana: Totowa, NJ, 2013; 10.1007/978-1-62703-550-7.

[ref21] Camelin E.; Romero O.; Piumetti M.; Ottone C.; Illanes A.; Fino D. Mechanisms of Interaction among Enzymes and Supports. In Nanomaterials for biocatalysis; Elsevier, 2021; pp 105–148, 10.1016/B978-0-12-824436-4.00022-8.

[ref22] Roik N. V.; Belyakova L. A. Sol-Gel Synthesis of MCM-41 Silicas and Selective Vapor-Phase Modification of Their Surface. J. Solid State Chem. 2013, 207, 194–202. 10.1016/j.jssc.2013.09.027.

[ref23] Pietricola G.; Tommasi T.; Dosa M.; Camelin E.; Berruto E.; Ottone C.; Fino D.; Cauda V.; Piumetti M. Synthesis and Characterization of Ordered Mesoporous Silicas for the Immobilization of Formate Dehydrogenase (FDH). Int. J. Biol. Macromol. 2021, 177, 261–270. 10.1016/j.ijbiomac.2021.02.114. 33621575

[ref24] Bradford M. M. A Rapid and Sensitive Method for the Quantitation of Microgram Quantities of Protein Utilizing the Principle of Protein-Dye Binding. Anal. Biochem. 1976, 72 (1–2), 248–254. 10.1016/0003-2697(76)90527-3. 942051

[ref25] Nesakumar N.; Thandavan K.; Sethuraman S.; Krishnan U. M.; Rayappan J. B. B. An Electrochemical Biosensor with Nanointerface for Lactate Detection Based on Lactate Dehydrogenase Immobilized on Zinc Oxide Nanorods. J. Colloid Interface Sci. 2014, 414, 90–96. 10.1016/j.jcis.2013.09.052. 24231089

[ref26] Pietricola G.; Ottone C.; Fino D.; Tommasi T. Enzymatic Reduction of CO2to Formic Acid Using FDH Immobilized on Natural Zeolite. J. CO2 Util. 2020, 42, 101343 10.1016/j.jcou.2020.101343.

[ref27] Lam K. F.; Chen X.; Mckay G.; Yeung K. L. Anion Effect on Cu 2 + Adsorption on NH 2 -MCM-41. Ind. Eng. Chem. Res. 2010, 47, 9376–9383. 10.1021/ie701748b.

[ref28] Lombardo M. V.; Videla M.; Calvo A.; Requejo F. G.; Soler-Illia G. J. A. A. Aminopropyl-Modified Mesoporous Silica SBA-15 as Recovery Agents of Cu(II)-Sulfate Solutions: Adsorption Efficiency, Functional Stability and Reusability Aspects. J. Hazard. Mater. 2012, 223–224, 53–62. 10.1016/j.jhazmat.2012.04.049. 22595542

[ref29] Cocuzza C.; Pietricola G.; Zonca I.; Dosa M.; Romero O.; Tommasi T.; Cauda V.; Fino D.; Ottone C.; Piumetti M. Simultaneous CO 2 Reduction and NADH Regeneration Using Formate and Glycerol Dehydrogenase Enzymes Co-Immobilized on Modified Natural Zeolite. RSC Adv. 2022, 12 (48), 31142–31155. 10.1039/D2RA03459J. 36349027 PMC9620777

[ref30] Alagöz D.; Toprak A.; Varan N. E.; Yildirim D.; Tükel S. S. Effective Immobilization of Lactate Dehydrogenase onto Mesoporous Silica. Biotechnol. Appl. Biochem. 2022, 69, 2550 10.1002/bab.2304. 34962677

[ref31] López-Gallego F.; Betancor L.; Mateo C.; Hidalgo A.; Alonso-Morales N.; Dellamora-Ortiz G.; Guisán J. M.; Fernández-Lafuente R. Enzyme Stabilization by Glutaraldehyde Crosslinking of Adsorbed Proteins on Aminated Supports. J. Biotechnol. 2005, 119 (1), 70–75. 10.1016/j.jbiotec.2005.05.021. 16039744

[ref32] Jackson E.; López-Gallego F.; Guisan J. M.; Betancor L. Enhanced Stability of L-Lactate Dehydrogenase through Immobilization Engineering. Process Biochem. 2016, 51 (9), 1248–1255. 10.1016/j.procbio.2016.06.001.

[ref33] Rocha-Martín J.; Rivas B. d. L.; Muñoz R.; Guisán J. M.; López-Gallego F. Rational Co-Immobilization of Bi-Enzyme Cascades on Porous Supports and Their Applications in Bio-Redox Reactions with Insitu Recycling of Soluble Cofactors. ChemCatChem 2012, 4 (9), 1279–1288. 10.1002/cctc.201200146.

[ref34] Piumetti M. Molecular Dynamics and Complexity in Catalysis and Biocatalysis 2022, 10.1007/978-3-030-88500-7.

[ref35] Piumetti M.; Illanes A. Enzymes and Their Function. In Molecular Dynamics and Complexity in Catalysis and Biocatalysis; Springer International Publishing: Cham, 2022; pp 23–53, 10.1007/978-3-030-88500-7_2.

[ref36] Granchi C.; Roy S.; Giacomelli C.; MacChia M.; Tuccinardi T.; Martinelli A.; Lanza M.; Betti L.; Giannaccini G.; Lucacchini A.; Funel N.; León L. G.; Giovannetti E.; Peters G. J.; Palchaudhuri R.; Calvaresi E. C.; Hergenrother P. J.; Minutolo F. Discovery of N-Hydroxyindole-Based Inhibitors of Human Lactate Dehydrogenase Isoform A (LDH-A) as Starvation Agents against Cancer Cells. J. Med. Chem. 2011, 54 (6), 1599–1612. 10.1021/jm101007q. 21332213

[ref37] Tang P.; Xu J.; Oliveira C. L.; Li Z. J.; Liu S. A Mechanistic Kinetic Description of Lactate Dehydrogenase Elucidating Cancer Diagnosis and Inhibitor Evaluation. J. Enzyme Inhib. Med. Chem. 2017, 32 (1), 564–571. 10.1080/14756366.2016.1275606. 28114833 PMC6010104

[ref38] Mercer J. M. Cooperativity. In Brenner’s Encyclopedia of Genetics, 2nd ed.; Elsevier: 2013; Vol. 2, pp 183–187, 10.1016/B978-0-12-374984-0.00339-9.

[ref39] Cornish-Bowden A.; Cárdenas M. L. Specificity of Non-Michaelis-Menten Enzymes: Necessary Information for Analyzing Metabolic Pathways. J. Phys. Chem. B 2010, 114 (49), 16209–16213. 10.1021/jp106968p. 21028874

[ref40] Pasti A. P.; Rossi V.; Di Stefano G.; Brigotti M.; Hochkoeppler A. Human Lactate Dehydrogenase A Undergoes Allosteric Transitions under PH Conditions Inducing the Dissociation of the Tetrameric Enzyme. Biosci. Rep. 2022, 42, BSR20212654 10.1042/BSR20212654. 35048959 PMC8799922

[ref41] Muttakin M.; Mitra S.; Thu K.; Ito K.; Saha B. B. Theoretical Framework to Evaluate Minimum Desorption Temperature for IUPAC Classified Adsorption Isotherms. Int. J. Heat Mass Transfer 2018, 122, 795–805. 10.1016/j.ijheatmasstransfer.2018.01.107.

[ref42] Otalvaro J. O.; Avena M.; Brigante M. Adsorption of Organic Pollutants by Amine Functionalized Mesoporous Silica in Aqueous Solution. Effects of PH, Ionic Strength and Some Consequences of APTES Stability. J. Environ. Chem. Eng. 2019, 7 (5), 103325 10.1016/j.jece.2019.103325.

[ref43] Ruiz-Cañas M. C.; Corredor L. M.; Quintero H. I.; Manrique E.; Romero Bohórquez A. R. Morphological and Structural Properties of Amino-Functionalized Fumed Nanosilica and Its Comparison with Nanoparticles Obtained by Modified Stöber Method. Molecules 2020, 25 (12), 2868 10.3390/molecules25122868. 32580500 PMC7355829

[ref44] Mercier L.; Pinnavaia T. J. Heavy Metal Ion Adsorbents Formed by the Grafting of a Thiol Functionality to Mesoporous Silica Molecular Sieves: Factors Affecting Hg(II) Uptake. Environ. Sci. Technol. 1998, 32 (18), 2749–2754. 10.1021/es970622t.

[ref45] Borrego T.; Andrade M.; Pinto M. L.; Rosa Silva A.; Carvalho A. P.; Rocha J.; Freire C.; Pires J. Physicochemical Characterization of Silylated Functionalized Materials. J. Colloid Interface Sci. 2010, 344 (2), 603–610. 10.1016/j.jcis.2010.01.026. 20129614

[ref46] Al-Dhrub A. H. A.; Sahin S.; Ozmen I.; Tunca E.; Bulbul M. Immobilization and Characterization of Human Carbonic Anhydrase I on Amine Functionalized Magnetic Nanoparticles. Process Biochem. 2017, 57 (March), 95–104. 10.1016/j.procbio.2017.03.025.

[ref47] Majoul N.; Aouida S.; Bessaïs B. Progress of Porous Silicon APTES-Functionalization by FTIR Investigations. Appl. Surf. Sci. 2015, 331, 388–391. 10.1016/j.apsusc.2015.01.107.

[ref48] Ferreira R. B.; Da Silva C. R.; Pastore H. O. Aminopropyl-Modified Magnesium-Phyllosilicates: Layered Solids with Tailored Interlayer Access and Reactivity. Langmuir 2008, 24 (24), 14215–14221. 10.1021/la802142s. 19360966

[ref49] Ataollahi N.; Cappelletto E.; Vezzù K.; Di Noto V.; Cavinato G.; Callone E.; Dirè S.; Scardi P.; Di Maggio R. Properties of Anion Exchange Membrane Based on Polyamine: Effect of Functionalized Silica Particles Prepared by Sol-Gel Method. Solid State Ionics 2018, 322 (May), 85–92. 10.1016/j.ssi.2018.04.022.

[ref50] Vittoni C.; Gatti G.; Paul G.; Mangano E.; Brandani S.; Bisio C.; Marchese L. Non-Porous versus Mesoporous Siliceous Materials for CO2 Capture. ChemistryOpen 2019, 8 (6), 719–727. 10.1002/open.201900084. 31275793 PMC6587325

[ref51] Yusdy; Patel S. R.; Yap M. G. S.; Wang D. I. C. Immobilization of L-Lactate Dehydrogenase on Magnetic Nanoclusters for Chiral Synthesis of Pharmaceutical Compounds. Biochem. Eng. J. 2009, 48 (1), 13–21. 10.1016/j.bej.2009.07.017.

[ref52] Braham S. A.; Siar E. H.; Arana-Peña S.; Bavandi H.; Carballares D.; Morellon-Sterling R.; de Andrades D.; Kornecki J. F.; Fernandez-Lafuente R. Positive Effect of Glycerol on the Stability of Immobilized Enzymes: Is It a Universal Fact?. Process Biochem. 2021, 102, 108–121. 10.1016/j.procbio.2020.12.015. PMC7918437 33673063

[ref53] Carbonaro M.; Nucara A. Secondary Structure of Food Proteins by Fourier Transform Spectroscopy in the Mid-Infrared Region. Amino Acids 2010, 38 (3), 679–690. 10.1007/s00726-009-0274-3. 19350368

[ref54] Morhardt C.; Ketterer B.; Heißler S.; Franzreb M. Direct Quantification of Immobilized Enzymes by Means of FTIR ATR Spectroscopy - A Process Analytics Tool for Biotransformations Applying Non-Porous Magnetic Enzyme Carriers. J. Mol. Catal. B Enzym. 2014, 107, 55–63. 10.1016/j.molcatb.2014.05.018.

[ref55] Parandi E.; Safaripour M.; Mosleh N.; Saidi M.; Rashidi Nodeh H.; Oryani B.; Rezania S. Lipase Enzyme Immobilized over Magnetic Titanium Graphene Oxide as Catalyst for Biodiesel Synthesis from Waste Cooking Oil. Biomass and Bioenergy 2023, 173, 106794 10.1016/j.biombioe.2023.106794.

[ref56] Xie W.; Zang X. Immobilized Lipase on Core-Shell Structured Fe3O4-MCM-41 Nanocomposites as a Magnetically Recyclable Biocatalyst for Interesterification of Soybean Oil and Lard. Food Chem. 2016, 194, 1283–1292. 10.1016/j.foodchem.2015.09.009. 26471683

[ref57] Carlsson N.; Gustafsson H.; Thörn C.; Olsson L.; Holmberg K.; Åkerman B. Enzymes Immobilized in Mesoporous Silica: A Physical-Chemical Perspective. Adv. Colloid Interface Sci. 2014, 205, 339–360. 10.1016/j.cis.2013.08.010. 24112562

[ref58] Illanes A. Enzyme Biocatalysis: Principles and Applications; Springer, 2008; 10.1007/978-1-4020-8361-7.

