# Supplementary material for: Preparation of a Mesoporous Biosensor for Human Lactate Dehydrogenase for Potential Anticancer Inhibitor Screening
Source: ACS Biomater Sci Eng. 2023 Oct 19;9(11):6045–57. doi: 10.1021/acsbiomaterials.3c00582 (PMC10646870; doi:10.1021/acsbiomaterials.3c00582)
Supplement: Supplementary file 1 — ab3c00582_si_001.pdf [file ab3c00582_si_001.pdf]

Support Information for publication

Preparation of a mesoporous biosensor for human lactate dehydrogenase as potential anticancer inhibitor screening

Clarissa Cocuzza[a], *Elena Antoniono*[a], *Carminna Ottone*[b], *Valentina Cauda*[a], *Debora Fino*[a], *Marco Piumetti*[a]*

[a]Department of Applied Science and Technology, Politecnico di Torino, Corso Duca degli Abruzzi, 24, 10129 Turin, Italy.

[b]Escuela de Ingeniería Bioquímica, Pontificia Universidad Católica de Valparaíso, Av. Brasil 2085, Valparaíso, Chile.

*Corresponding author: marco.piumetti@polito.it

- Figure S1 showing the scheme of the functionalization process performed on MCM-41 to provide amino and aldehyde (glyoxyl) functional groups (page S3).

- Figure S2 showing the detailed scheme of the immobilization process of *h*LDH-A on MCM-41_{A}, preactivated with glutaraldehyde, and MCM-41_{AG} (page S4).

- Figure S3 showing the comparison between Lineweaver-Burk and Hanes-Woolf linearization models applied to the results of the kinetic tests (page S5).

- Figure S4 showing the evaluation of the amino groups present on the surface of the MCM-41 after the functionalization process. (page S5).

- Description of the procedure used for the evaluation of MCM-41 cell parameters (page S6).
- Figure S5 showing the optical fluorescence microscopy image acquired on MCM-41_{AG} (page S6).

- Figure S6 showing the X-ray diffractograms of MCM-41, MCM-41A, MCM-41AG, and imm-hLDH-A at low angles (2θ range 2° – 5°) (page S7).

- Table S1 listing the activity (%) results obtained as a function of pH and temperature (°C) for free and immobilized (page S7)

- Figure S7 showing a map of the pH and temperature conditions analyzed (page S8).

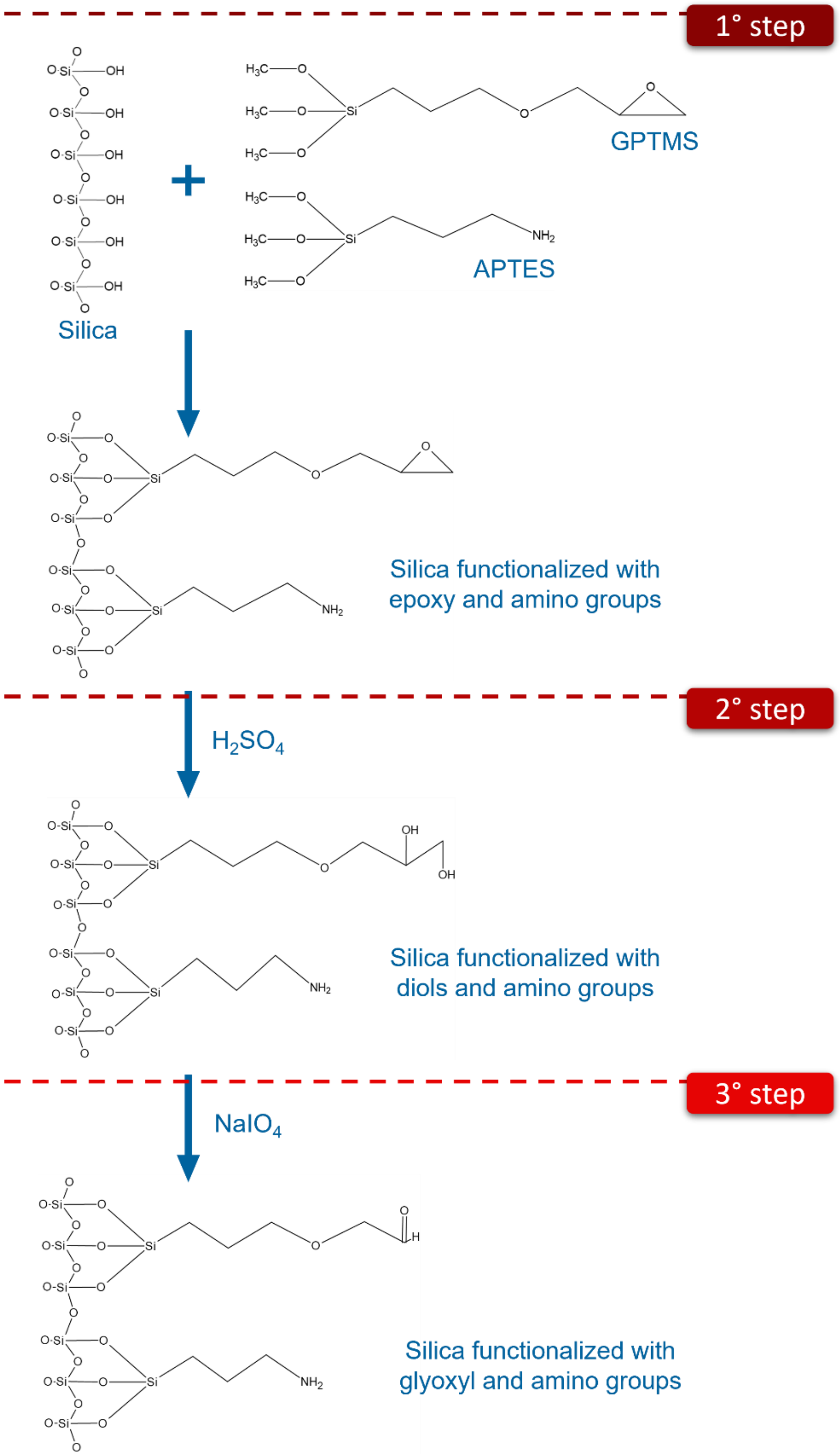

Figure S1. Scheme of the functionalization process to obtain amino and glyoxyl groups on MCM-41. To have MCM-41$_A$ the process is limited to the first step in the absence of GPTMS.

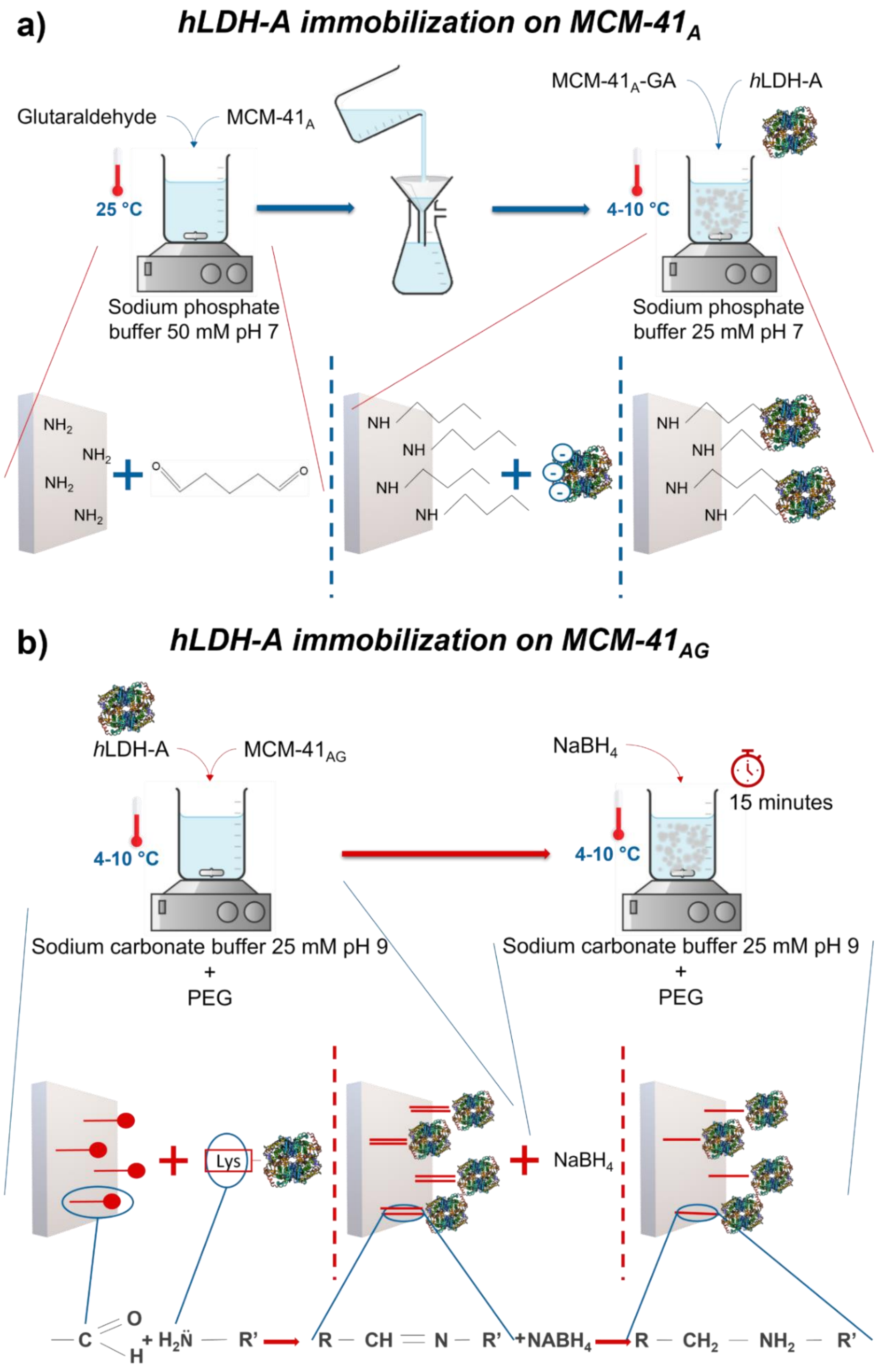

Figure S2. Scheme of the *h*LDH-A immobilization procedure on a) MCM-41$_A$ and b) MCM-41$_{AG}$

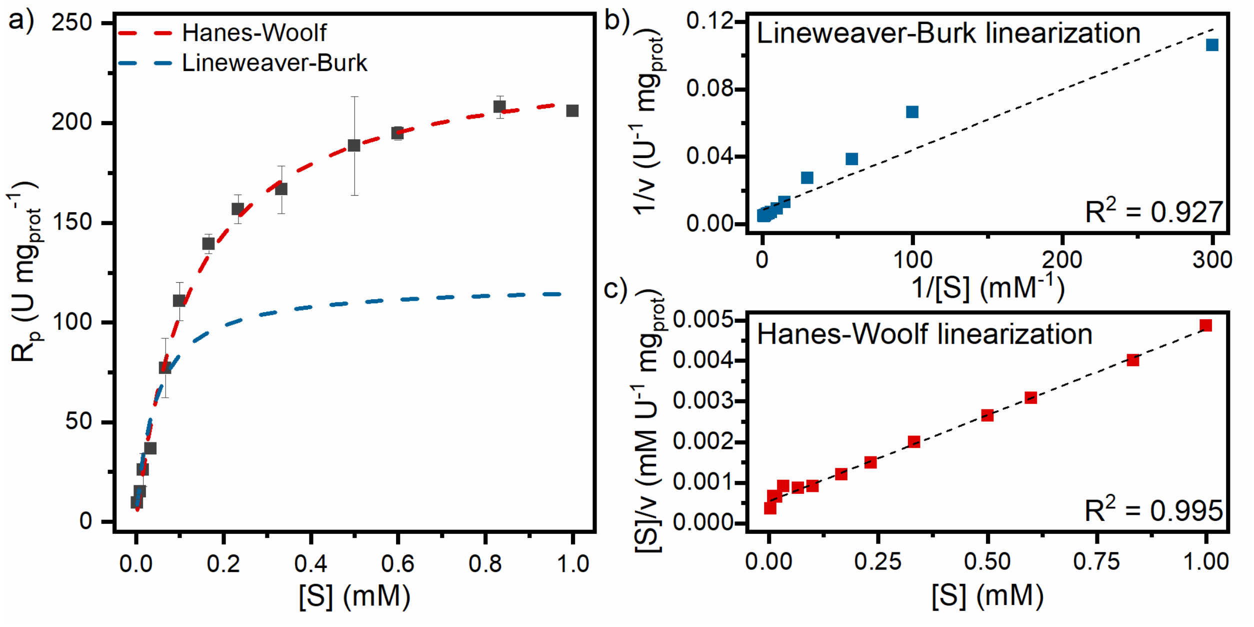

Figure S3. Kinetic tests outcomes of the pyruvate reduction, performed at 35 °C and pH 7.5 a) comparison of the models obtained with Lineweaver-Burk and Hanes-Woolf linearization, b) Lineweaver-Burk linearization and c) Hanes-Woolf linearization.

Quantification of amino groups. The MCM-41$_A$ (0.05 g) was put in a solution of $CuSO_4$ 300 mM (1.5 ml) and stirred for 1 hour at room temperature. The suspension was finally centrifuged, and the supernatant was diluted to be analyzed using UV-Vis spectroscopy.

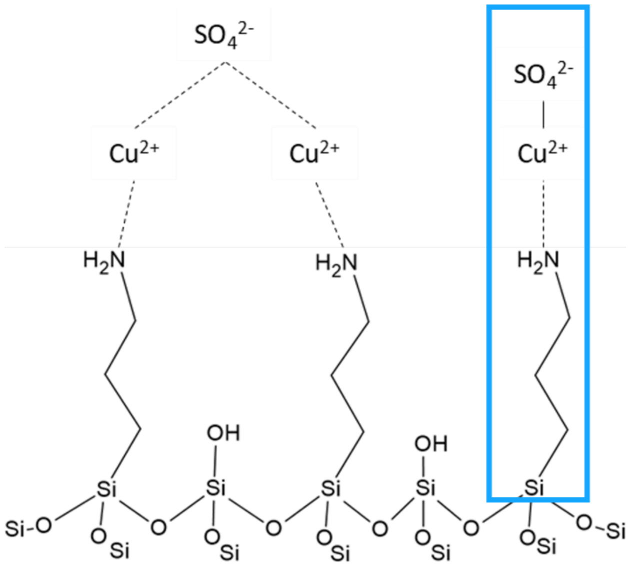

Figure S4. Schematization of possible interactions between amino groups and $CuSO_4$ [28].

Evaluation of MCM-41 cell parameters. The regular structure of MCM-41 enables calculating the cell parameters: inter-reticular distance (d_0), the cell parameter (a_0), and wall thickness (δ). The formula expressed in Equation (S1) can be used to calculate d_0, Equation (S2) to calculate a_0, and finally, Equation (S3) to calculate δ.

$$D_0=\frac{\lambda}{2\sin(\theta)} \quad \text{(S1)}$$

where λ is the $K_{\alpha 1}$ radiation of Cu (0.1540598 nm) and θ is half of the (100) peak position.

$$A_0=\frac{2d_0}{\sqrt{3}} \quad \text{(S2)}$$

from Equation (11) a_0 is estimated. The wall thickness is obtained by substituting the values of a_0 and d_{BJH} in Equation (12).

$$\delta=a_0-d_{BJH} \quad \text{(S3)}$$

The wall thickness was evaluated only for MCM-41 (δ=1.6 nm) because the modifications introduced by the functionalization process make unreliable the results obtained for MCM-41$_A$ and MCM-41$_{AG}$.

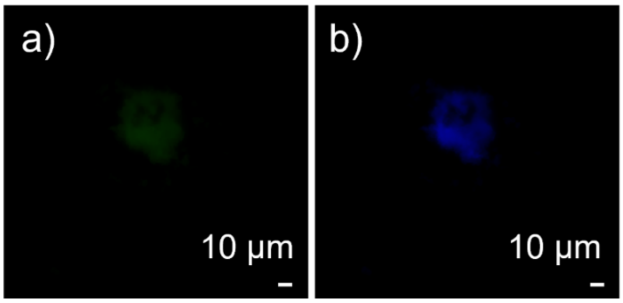

Figure S5. Optical fluorescence microscopy images of the MCM-41$_{AG}$. Image (a) depicts the green channel and image (b) depicts the blue channel, showing a slight fluorescence. The brightness and contrast of the images were corrected.

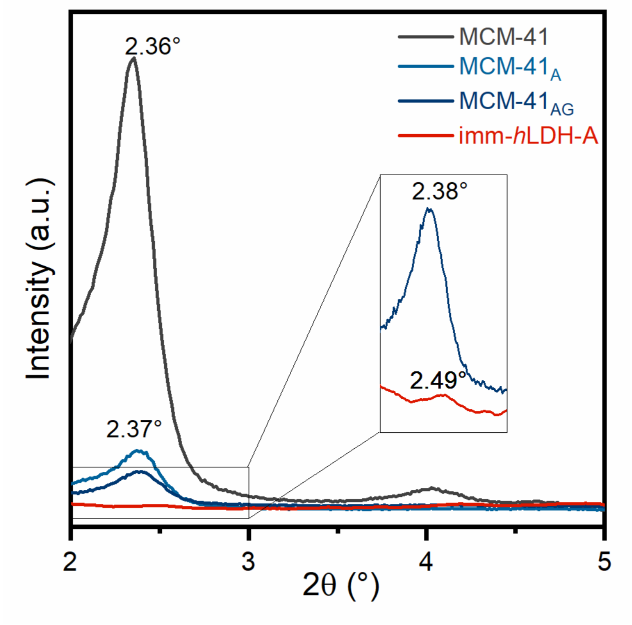

Figure S6. X-ray diffractograms of MCM-41, MCM-41$_A$, MCM-41$_{AG}$, and imm-*h*LDH-A at low angles (2θ range 2° – 5°)

Table S1. Activity obtained as a function of the different conditions of pH and temperature for the free and immobilized enzyme

pH	T (°C)	A_{free} (%)	$A_{immobilized}$ (%)
5	25	44	20
5	45	49	100
5	65	55	9
8	25	46	59
8	45	100	6
8	65	4	5
11	25	2	0
11	45	1	51
11	65	1	6

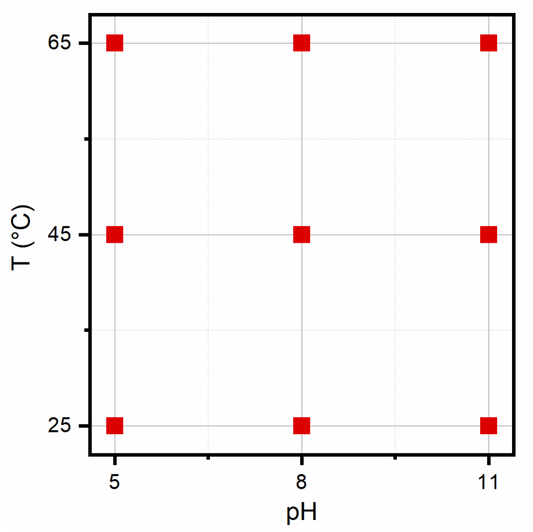

Figure S7. pH and temperature conditions tested (■) to obtain the pH and temperature profiles
